# Changes in antibiotic consumption, AMR and *Clostridioides difficile* infections in a large tertiary-care center following the implementation of institution-specific guidelines for antimicrobial therapy: A nine-year interrupted time series study

**DOI:** 10.1371/journal.pone.0258690

**Published:** 2021-10-14

**Authors:** Sebastian G. Schönherr, Donald Ranft, Norman Lippmann, Christoph Lübbert

**Affiliations:** 1 Division of Infectious Diseases and Tropical Medicine, Department of Medicine II, Leipzig University Hospital, Leipzig, Germany; 2 Hospital Pharmacy, Leipzig University Hospital, Leipzig, Germany; 3 Institute for Medical Microbiology and Virology, Leipzig University Hospital, Leipzig, Germany; 4 Interdisciplinary Center for Infectious Diseases (ZINF), Leipzig University Hospital, Leipzig, Germany; 5 Department of Infectious Diseases/Tropical Medicine, Nephrology and Rheumatology, Hospital St. Georg, Leipzig, Germany; School of Pathology, National Health Laboratory Service (NHLS) and University of the Witwatersrand, SOUTH AFRICA

## Abstract

**Background:**

Institution-specific guidelines (ISGs) within the framework of antimicrobial stewardship programs offer locally tailored decision support taking into account local pathogen and resistance epidemiology as well as national and international guidelines.

**Objectives:**

To assess the impact of ISGs for antimicrobial therapy on antibiotic consumption and subsequent changes in resistance rates and *Clostridioides difficile* infections (CDIs).

**Methods:**

The study was conducted at the Leipzig University Hospital, a 1,451-bed tertiary-care medical center, and covered the years 2012 to 2020. Since 2014, ISGs were provided to optimize empirical therapies, appropriate diagnostics, and antimicrobial prophylaxis. We used interrupted time series analysis (ITSA) and simple linear regression to analyze changes in antimicrobial consumption, resistance and CDIs.

**Results:**

Over the study period, 1,672,200 defined daily doses (DDD) of antibiotics were dispensed, and 85,645 bacterial isolates as well as 2,576 positive *C*. *difficile* cultures were collected. Total antimicrobial consumption decreased by 14% from 2012 to 2020, without clear impact of the deployment of ISGs. However, implementation of ISGs was associated with significant decreases in the use of substances that were rarely recommended (e.g., fluoroquinolones). Over the whole study period, we observed declining resistance rates to most antibiotic classes of up to 25% in *Enterobacterales*, staphylococci, and *Pseudomonas aeruginosa*. Switching from ceftriaxone to cefotaxime was associated with reduced resistance to third-generation cephalosporins. The number of CDI cases fell by 65%, from 501 in 2012 to 174 in 2020.

**Conclusions:**

Well-implemented ISGs can have a significant, immediate, and lasting impact on the prescription behavior. ISGs might thereby contribute to reduce resistance rates and CDI incidences in the hospital setting.

## Introduction

### Background

Antimicrobial resistance (AMR) is considered one of the greatest global health threats [1]. Multidrug-resistant infections are associated with longer hospital stays, higher treatment costs, and increased morbidity and mortality. In Europe alone, over 33,000 annual deaths are attributed to resistant infections [2,3]. Inappropriate and incorrect prescribing is a major driver for resistance and is associated with poorer outcomes [4–6]. In response to the increase in resistance, antimicrobial stewardship (AMS) programs have been widely implemented, aiming to optimize therapies and outcomes. Recent metanalyses showed that AMS programs were associated with enhanced appropriate use of antimicrobials, reduced incidences of AMR and *Clostridioides difficile* infections (CDIs), shorter hospital stays, and lower therapy costs [7–9].

Institution-specific guidelines (ISGs) are considered an important part of AMS programs, as they offer locally-tailored decision support for empirical therapies while reflecting local pathogen and resistance epidemiology as well as national and other societal recommendations [10,11]. Several studies showed that local guidelines can lead to behavioral changes among physicians, yet few investigated the influence on antimicrobial consumption and resistance [12–16]. The Leipzig University Hospital first introduced ISGs in 2014 as a printed pocket guide; an electronic application was added in 2017 [17,18]. In a previous, survey-based study we found that the ISGs were successfully implemented, regularly used across disciplines and had a perceived influence on physicians’ prescribing behavior [17].

### Objectives

In this study we aimed to assess the effects of ISGs on antimicrobial consumption and subsequently the incidence of AMR and CDIs. We hypothesized that, after the implementation of the ISGs, the use of antimicrobial agents that were commonly prescribed prior to the intervention but not often recommended in the ISGs (i.e., ciprofloxacin, sultamicillin, oral cefuroxime, ceftriaxone; **Table 1**) would be reduced. Subsequently, we expected a decrease in resistance to the respective antibiotics and a decrease in CDI. Conversely, we expected an increased use of antibiotic agents that were not used frequently before the introduction of the ISGs but were recommended frequently, such as cefotaxime, or oral clarithromycin (combination partner for the treatment of community-acquired pneumonia). Furthermore, we aimed to assess whether there would be secondary effects on the proportion of oral and parenteral antibiotics use, as well as on the proportions of Access, Watch and Reserve antibiotics as defined by the World Health Organization (WHO) AWaRe classification [19].

**Table 1:** ISG recommendations for commonly prescribed antibiotics and hypothesis regarding changes in prescription based on the number of recommendations and consumption in 2012.

Antibiotic agent (application)	Percent of total antibiotic consumption		Number of recommendations in the ISGs including combination partners		Relevant first line recommendation for empirical therapy (number of first line recommendations, including combination partners)	Hypothesis (+ = expected increase,— = expected decrease)	Difference of DDD/100BD between 2012 and 2020, in percent of the value for 2012
	2012	2020	First-line	Alternative			
**Ciprofloxacin (O)**	14%	5%	4	1	Infectious enterocolitis; epidydimitis and prostatitis; HAP; neutropenic fever (low risk); otitis externa maligna	-	-68%
**Cefuroxime (O)**	9%	3%	2	2	Facial boil; UTI in pregnancy	-	-74%
**Ceftriaxone (P)**	4%	1%	2	2	Acute cholecystitis; gonorrhoea	-	-79%
**Cefuroxime (P)**	5%	5%	2	2	Perioperative prophylaxis	-	-16%
**Cefotaxime (P)**	1%	14%	7	4	Abdominal infections (2); complicated UTI; post-operative infections; community-acquired sepsis; community-acquired meningitis; mastoiditis	+	+829%
**Sultamicillin (O)**	6%	2%	1	1	Neutropenic fever (low risk); bite injury	-	-71%
**Ampicillin/sulbactam (P)**	4%	6%	16	5	CAP; skin and soft tissue infections (2); ENT infections (10); maxillofacial surgery (2); neutropenic fever (low risk); perioperative prophylaxis	ND	+13%
**Piperacillin/tazobactam (P)**	5%	7%	7	11	Secondary peritonitis; necrotizing fasciitis; complicated airway infection including HAP (4)	ND	+29%
**Imipenem (P)**	5%	2%	2	8	Neutropenic fever (intermediate and high risk)	-	-65%
**Meropenem (P)**	2%	5%	1	6	Nosocomial meningitis	-	+91%
**Vancomycin (P,O)**	5%	4%	3	2	CDI (oral)*; CRSBI; nosocomial meningitis	ND	-27%
**Metronidazole (P)**	3%	2%	4	4	Abdominal infections (3); adnexitis; perioperative prophylaxis	ND	-23%
**Metronidazole (O)**	2.5%	0.5%	4	4	Giardiasis	-	-84%
**Trimethoprim/sulfamethoxazole (O)**	3%	3.5%	1	0	PCP	-	+3%
**Clindamycin (P)**	2%	4%	8	12	Skin and soft tissue infections (4); bone and joint infections (5); bite injury	+	+72%
**Clarithromycin (O)**	0.5%	4%	2	0	CAP	+	+2%

**Abbreviations:** (P) = parenteral, (O) = oral, HAP = hospital-acquired pneumonia, CAP = community-acquired pneumonia, CDI = *Clostridioides difficile* infection, CRSBI = catheter-related bloodstream infections, ENT = ear nose throat, UTI = urinary tract infection, PCP = pneumocystis pneumonia, ND = not to be determined.

*Vancomycin is administered orally for the treatment of CDIs but accounted as parenteral since it is dispensed in the same formulation.

## Methods

### Study design

We conducted an observational interrupted time series study with antibiotic consumption, resistance and CDI data to assess the effect of the ISGs. Simple and segmented linear regression models were used to analyze trends and changes thereof. The study is reported in accordance with the STROBE-AMS recommendations of the EQUATOR network [20,21].

### Setting

The Leipzig University Hospital (LUH) is a 1,451-bed tertiary-care facility with 29 departments and clinics of all specialties in Leipzig, Germany. Since 2012, several interdisciplinary AMS interventions have been implemented, including regular ward visits by the AMS team, intense training of staff, restriction of selected reserve antibiotics, surveillance of antibiotic consumption and resistance and the implementation of professional ISGs, as described before [17,22–24].

### Intervention

The ISGs were developed in cooperation with experts from the represented disciplines and implemented in June 2014. They cover a wide range of infections for many clinical disciplines and provide information on empirical treatment, diagnostics and prophylaxis as well as dose adjustments. The current 4th edition of the ISGs (in German) can be accessed online [18].

### Participants

All inpatient departments, except pediatrics and psychiatry, were included in the analyses. Psychiatric units were excluded as very few antimicrobials are prescribed there, and pediatric units as they prescribe at small absolute doses. Their inclusion could have led to an underestimation of the antibiotic consumption in the hospital overall. Outpatient departments were excluded since the ISGs are specifically designed for the inpatient setting.

### Microbiology

The microbiological analysis focused on isolates from *Klebsiella pneumoniae*, *Escherichia coli*, *Staphylococcus aureus*, *S*. *epidermidis*, *Enterococcus faecalis*, *E*. *faecium* and *Pseudomonas aeruginosa* collected 2012 to 2020. All diagnostic samples from the entire hospital with the exception of routine screening material were included in the analysis. In addition, all CDIs confirmed by positive cultures were recorded.

### Variables

Antibiotic consumption was measured in monthly defined daily doses (DDD) per 100 bed days (DDD/100BD). We focused on antibiotic agents and antibiotic combinations that met at least one of the following criteria: 1) they were among the 15 most frequently dispensed antibiotics in the year before the intervention; 2) they were frequently recommended as first-line therapy in the ISGs; 3) they received broad recommendation for at least one very common infection (**Table 1**). The analysis was done separately for parenteral and oral application. Furthermore, we analyzed relevant commonly used groups (**Table 2**).

**Table 2:** Results of the interrupted time series analysis of changes in dispensation of antimicrobials in the LUH between 2012 and 2020, associated with the introduction of ISGs and other antibiotic-specific interventions. For fluoroquinolones: Two warning letters [“Rote-Hand-Briefe”] issued by the German Federal Institute for Drugs and Medical Devices (BfArM) in October 2018 and April 2019 warning about serious adverse effects of fluoroquinolones; for trimethoprim/sulfamethoxazole: The introduction of routine real-time PCR for the diagnosis of pneumocystis pneumonia [PCP]). Psychiatric units, pediatric units and outpatient departments were excluded from the analysis.

Antibiotic agent (application)	Baseline antibiotic consumption in DDD/100BD (95%CI) (β0)	Baseline trend in DDD/100BD per month (95%CI)	Level change one month after the intervention in DDD/100BD (95%CI)	Trend change after the intervention in DDD/100BD per month (95%CI)	Level change one month after the second intervention in DDD/100BD (95%CI)	Trend change after the second intervention in DDD/100BD per month (95%CI)
**Total antibiotic consumption**						
Antibiotics	46.9 (44.5 to 49.3) ***	-0.23 (-0.37 to -0.1) **	-0.9 (-3.7 to 1.8)	0.23 (0.09 to 0.37) **		
Antibiotics (P)	24.5 (22.9 to 26) ***	-0.07 (-0.16 to 0.01)	-1 (-2.7 to 0.8)	0.16 (0.07 to 0.25) ***		
Antibiotics (O)	22.3 (21.1 to 23.6) ***	-0.16 (-0.23 to -0.09) ***	0.1 (-1.3 to 1.5)	0.07 (-0.01 to 0.14)		
**WHO AWaRe Classification**						
Access	16.9 (15.9 to 17.9) ***	-0.1 (-0.16 to -0.04) ***	-1.8 (-2.9 to -0.7) **	0.13 (0.08 to 0.19) ***		
Watch	30.3 (28.6 to 32) ***	-0.16 (-0.26 to -0.06) **	0.9 (-1 to 2.9)	0.13 (0.03 to 0.23) **		
Reserve	0.9 (0.7 to 1.1) ***	0.02 (0.01 to 0.03) ***	-0.3 (-0.6 to -0.1) **	-0.01 (-0.02 to 0)		
**Penicillins**	9.6 (8.8 to 10.4) ***	-0.01 (-0.06 to 0.04)	-0.6 (-1.6 to 0.3)	0.04 (-0.01 to 0.09)		
Penicillin/beta-lactam inhibitor combinations	7.1 (6.6 to 7.6) ***	-0.03 (-0.05 to 0.00)	-1.0 (-1.5 to -0.4) ***	0.03 (0.00 to 0.06)		
Ampicillin/sulbactam (P)	1.9 (1.7 to 2.2) ***	0.01 (0 to 0.02)	-0.4 (-0.7 to -0.2) ***	0.0 (-0.01 to 0.01)		
Sultamicillin (O)	2.9 (2.7 to 3.1) ***	-0.04 (-0.05 to -0.02) ***	-0.4 (-0.7 to -0.2) ***	0.03 (0.01 to 0.04) ***		
Piperacillin/tazobactam (P)	2.2 (2 to 2.5) ***	0 (-0.01 to 0.02)	0 (-0.3 to 0.3)	0 (-0.01 to 0.02)		
**Cephalosporins**	10.7 (10 to 11.4) ***	-0.07 (-0.11 to -0.03) **	-0.1 (-0.9 to 0.7)	0.08 (0.04 to 0.12) ***		
2G Cephalosporins	6.6 (6.1 to 7.1) ***	-0.03 (-0.06 to 0)	0.1 (-0.5 to 0.7)	-0.01 (-0.04 to 0.02)		
3G Cephalosporins	3.7 (3.2 to 4.1) ***	-0.03 (-0.05 to 0) *	-0.2 (-0.7 to 0.3)	0.07 (0.05 to 0.1) ***		
Cefotaxime (P)	0.5 (0.2 to 0.8) ***	0.01 (-0.01 to 0.02)	0.2 (-0.2 to 0.5)	0.06 (0.04 to 0.07) ***		
Ceftriaxone (P)	1.9 (1.8 to 2.1) ***	-0.03 (-0.03 to -0.02) ***	-0.3 (-0.5 to -0.1) ***	0.02 (0.01 to 0.03) ***		
Cefuroxime (O)	4.1 (3.7 to 4.6) ***	-0.02 (-0.05 to 0) *	0.1 (-0.4 to 0.5)	-0.01 (-0.03 to 0.02)		
Cefuroxime (P)	2.4 (2.2 to 2.6) ***	0 (-0.02 to 0.01)	0.1 (-0.2 to 0.3)	0 (-0.01 to 0.01)		
**Fluoroquinolones**	9.2 (8.6 to 9.8) ***	-0.06 (-0.09 to -0.03) **	-0.9 (-1.6 to -0.1) *	0.04 (0.01 to 0.08) *	-1.7 (-2.5 to -0.9) ***	-0.04 (-0.09 to 0)
Ciprofloxacin	6.5 (6.1 to 6.9) ***	-0.03 (-0.05 to 0) *	-1.1 (-1.6 to -0.6) ***	0.01 (-0.01 to 0.04)	-1.1 (-1.7 to -0.5) ***	-0.02 (-0.05 to 0.01)
Moxifloxacin	1.1 (0.9 to 1.3) ***	-0.01 (-0.02 to 0)	0 (-0.2 to 0.2)	0 (-0.01 to 0.01)	-0.1 (-0.3 to 0.1)	0.01 (-0.01 to 0.02)
Levofloxacin	1.6 (1.3 to 1.8) ***	-0.02 (-0.04 to -0.01) **	0.2 (-0.1 to 0.5)	0.03 (0.01 to 0.05) **	-0.5 (-0.8 to -0.1) **	-0.03 (-0.05 to -0.01) *
**Carbapenems**	3.6 (3.3 to 3.9) ***	-0.01 (-0.02 to 0.01)	-0.1 (-0.4 to 0.3)	0 (-0.02 to 0.02)		
Meropenem (P)	1.1 (0.8 to 1.3) ***	0.01 (-0.01 to 0.02)	0 (-0.3 to 0.2)	0 (-0.01 to 0.02)		
Imipenem (P)	2.5 (2.3 to 2.7) ***	-0.01 (-0.02 to 0) *	0 (-0.3 to 0.2)	0 (-0.01 to 0.01)		
**Other antibiotic agents**						
Trimethoprim/sulfamethoxazole	1.37 (1.17 to 1.57) ***	-0.01 (-0.02 to 0.01)	-0.43 (-0.73 to -0.12) **	0.01 (-0.01 to 0.03)	-0.02 (-0.28 to 0.25)	0.01 (-0.01 to 0.02)
Clarithromycin (O)	1.8 (1.5 to 2.1) ***	-0.02 (-0.04 to 0)	0.8 (0.4 to 1.1) ***	0.01 (-0.01 to 0.03)		
Clindamycin	2 (1.6 to 2.3) ***	0.01 (0 to 0.03)	-0.1 (-0.4 to 0.3)	0 (-0.02 to 0.02)		
Vancomycin	2.3 (2 to 2.6) ***	-0.04 (-0.05 to -0.02) ***	0.4 (0.1 to 0.7) **	0.04 (0.02 to 0.05) ***		
Metronidazole (P)	1.3 (1.1 to 1.4) ***	-0.01 (-0.02 to 0) *	-0.1 (-0.3 to 0)	0.01 (0 to 0.02) *		
Metronidazole (O)	1.3 (1.1 to 1.4) ***	-0.01 (-0.02 to -0.01) ***	-0.1 (-0.2 to 0)	0 (0 to 0.01)		

**Abbreviations:** (P) = parenteral, (O) = oral.

* = p < 0.05

** = p < 0.01

*** = p < 0.001.

Resistance outcomes were measured annually in percent of total isolates. We used the 2021 EUCAST breakpoints (www.eucast.org) to determine annual resistance rates [25]. Klicken oder tippen Sie hier, um Text einzugeben. Intermediate (susceptible, increased exposure) minimum inhibitory concentrations (MICs) were not considered resistant. The absolute number of positive *C*. *difficile* routine cultures was used to analyze CDI activity.

### Data sources

The hospital pharmacy provided data on antimicrobials dispensed to hospital cost centers (e.g. wards) for the period from 1 January 2012 to 31 December 2020. The data was accessed via the hospital benchmarking software IQVIA Premax (www.iqvia.com). Monthly bed days (BD) of all cost centers were received from the controlling department. Annual MIC data of pathogen isolates and the number of CDIs were provided by the Institute for Medical Microbiology and Virology. German antibiotic resistance data collected in the European Antimicrobial Resistance Network (EARS-Net) was accessed through the ECDC Surveillance Atlas for AMR (www.ecdc.europa.eu/en/antimicrobial-resistance/surveillance-and-disease-data/data-ecdc) [26].

### Addressing biases—Exclusion and sub-setting of data

We considered regular ward rounds by the AMS team and interventions by other departments as strong potential confounders. To address this, we ran all analyses again with a subset that did not include wards receiving regular AMS rounds (i.e., intensive care units, pneumology, gastroenterology, and two surgical wards) and hematologic oncology wards, as these wards have their own antimicrobial therapy recommendations.

A stockout of ampicillin/sulbactam caused a drastic reduction of its use between September 2015 and February 2016; to avoid this as a potential confounder in the analysis we excluded the respective data points in this timeframe [27]. A change in the record keeping system of BD in 2013 caused a large increase in BD (about 40%) without any relevant change of activity in the hospital. To avoid a confounding effect on the outcome variable (DDD/100BD), we adjusted the BD between January 2012 and July 2013 with a correcting factor (1.38) that was determined by comparing the monthly averages of a five-month period that was fully documented in both records (August to December 2013). Old, new, and adjusted monthly BD are plotted in **S1 Fig**.

Hospital resistance rates are partly determined by external factors such as treatment in other facilities or the spread of resistant strains in the population, and observed changes in resistance rates are therefore not necessarily due to changes in prescribing within the hospital. We therefore also compared our data to selected resistance rates collected in the EARS-Net, to control for broad changes in resistance rates within the German patient population [26]. The documented *S*. *aureus* resistance rates in the LUH were extremely high in 2014, likely due to a reporting or documentation error, resulting in a strong negative trend in the post-intervention phase. To avoid misleading results, we did not include 2014 *S*. *aureus* resistance rates in the analysis.

### Statistical methods

A segmented linear regression model of interrupted time series was used to analyze the changes in antibiotic consumption, as described before [28–31]. This method allows for the evaluation of immediate changes in level, as well as long-term effects due to trend changes. A time series is a sequence of observations of a certain outcome, which is aggregated for each time point (e.g. DDD/100BD per month). The time series is divided (interrupted) into pre- and post-intervention segments. Least square regression lines are fitted to each segment of the time series and together expressed by the following model [28]:

$$Y_{t}=\beta 0+\beta 1*time_{t}+\beta 2*intervention_{t}+\beta 3*time\;after\;intervention_{t}+e_{t.}$$

In this model, Y_t_ is the outcome at time point t, in this case DDD/100BD in each month. *Time* is a continuous variable counting the number of months of the whole timeframe, *intervention* is an indicator for the time before (*intervention* = 0) and after (*intervention* = 1) the intervention, and *time after intervention* is a continuous variable counting the time after the intervention. β0 estimates the baseline level at time point zero (y intercept), β1 estimates the baseline trend, β2 estimates the level change between the pre- and the post-intervention period, and β3 estimates the change in trend after the intervention. The sum of β1 and β3 estimates the post-intervention slope. The error e_t_ represents the variability not described by the model.

Since the ISGs were implemented in June 2014, July 2014 was chosen as the first time point of the post-intervention period. A second intervention was added in two constellations. First, on 26 June 2016 the microbiology laboratory introduced routine real-time polymerase chain reaction (PCRs) for the diagnosis of pneumocystis pneumonia (PCP). This led to a higher detection rate, and subsequently more prescriptions of trimethoprim/sulfamethoxazole. We considered this a second intervention for trimethoprim/sulfamethoxazole, and so July 2016 was set as the first post-intervention data point. Second, two warning letters (“Rote-Hand-Briefe”) had been issued by the German Federal Institute for Drugs and Medical Devices (BfArM) in October 2018 and April 2019, warning of the serious adverse effects of fluoroquinolones [32,33]. These warnings were actively conveyed to hospital physicians by the AMS team and reflected in their recommendations. November 2018 was chosen as the first post-intervention data point.

MIC data was only available in annual aggregates which means that there were not enough data points to run ITSA. We calculated simple linear regressions to determine the trends of resistance rates over the whole study period. To assess national trends of AMR we used annual resistance rates from 2012 to 2019, as data for 2020 was not uploaded at the time of submission [26]. P values of <0.05 were considered statistically significant.

All calculations were carried out using RStudio [34]. The R-code will be shared on request.

### Ethical considerations

The ethics committee of the Medical Faculty of Leipzig University reviewed and approved the study on January 22, 2019 (registry number 011/19-ek). All data is anonymous.

## Results

The LUH dispensed a total of 1,672,200 DDD of antibiotics during the study period (excluding pediatric and psychiatric units and outpatient services). The antibiotic groups prescribed most over the study period were penicillins, accounting for 24% of total antibiotic DDD, followed by cephalosporins (23%) and fluoroquinolones (15%). The antibiotic agents with the highest overall consumption were cefuroxime (12%), ciprofloxacin (10%), and ampicillin/sulbactam, including sultamicillin (8%). A total of 4,192,497 adjusted BD were documented. Monthly BD rose slowly over the study period without major changes (**S1 Fig**). We tested 85,645 isolates of eight pathogens regarding their resistance and collected 2,576 positive *C*. *difficile* cultures over the study period.

### Antibiotic consumption

**Fig 1:**
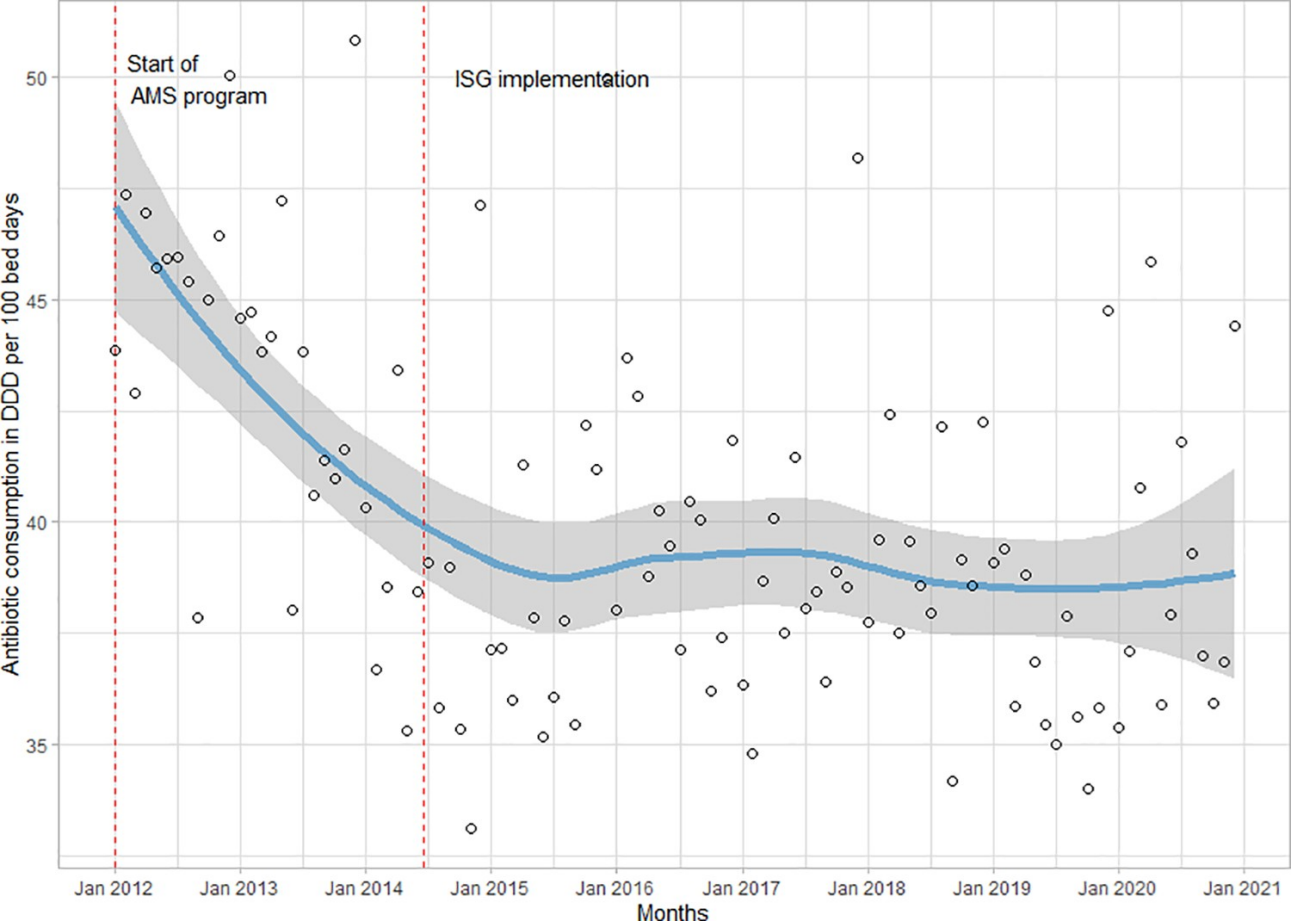
Total antibotic consumption in the LUH between 2012 and 2020. Plotted in monthly defined daily doses per 100 bed days (DDD/100BD). Dashed lines represent the start of the antimicrobial stewardship (AMS) program and the implementation of ISGs.

Between 2012 and 2020 the annual antibiotic consumption per 100 BD fell by 14%, from 543 DDD/100BD to 468 DDD/100BD. This reduction mainly occurred prior to the study intervention (**Fig 1**).

**Table 2** shows the results of the ITSA of total antibiotic consumption as well as of different subgroups and antibiotic agents.

**Fig 2:**
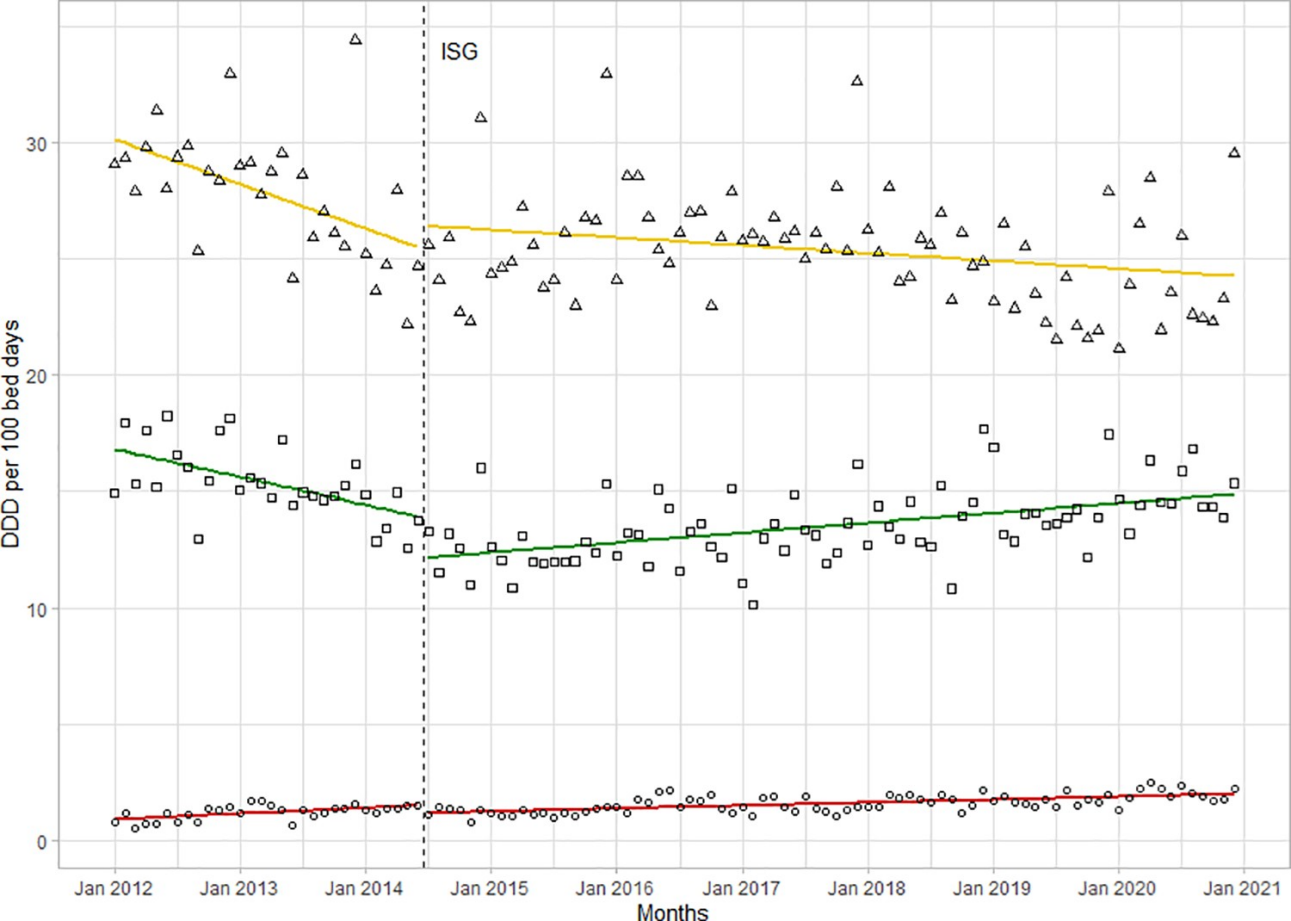
Consumption of Access (triangle), Watch (square) and Reserve (circle) antibiotics. As defined by the 2019 WHO AWaRe Classification in the LUH before and after the introduction of ISGs. Squares represent Access antibiotics, triangles represent Watch antibiotics, circles represent Reserve antibiotics, dashed line represents the introduction of ISGs.

Changes in the proportions of AWaRe groups were minimal (**Fig 2**).

**Fig 3:**
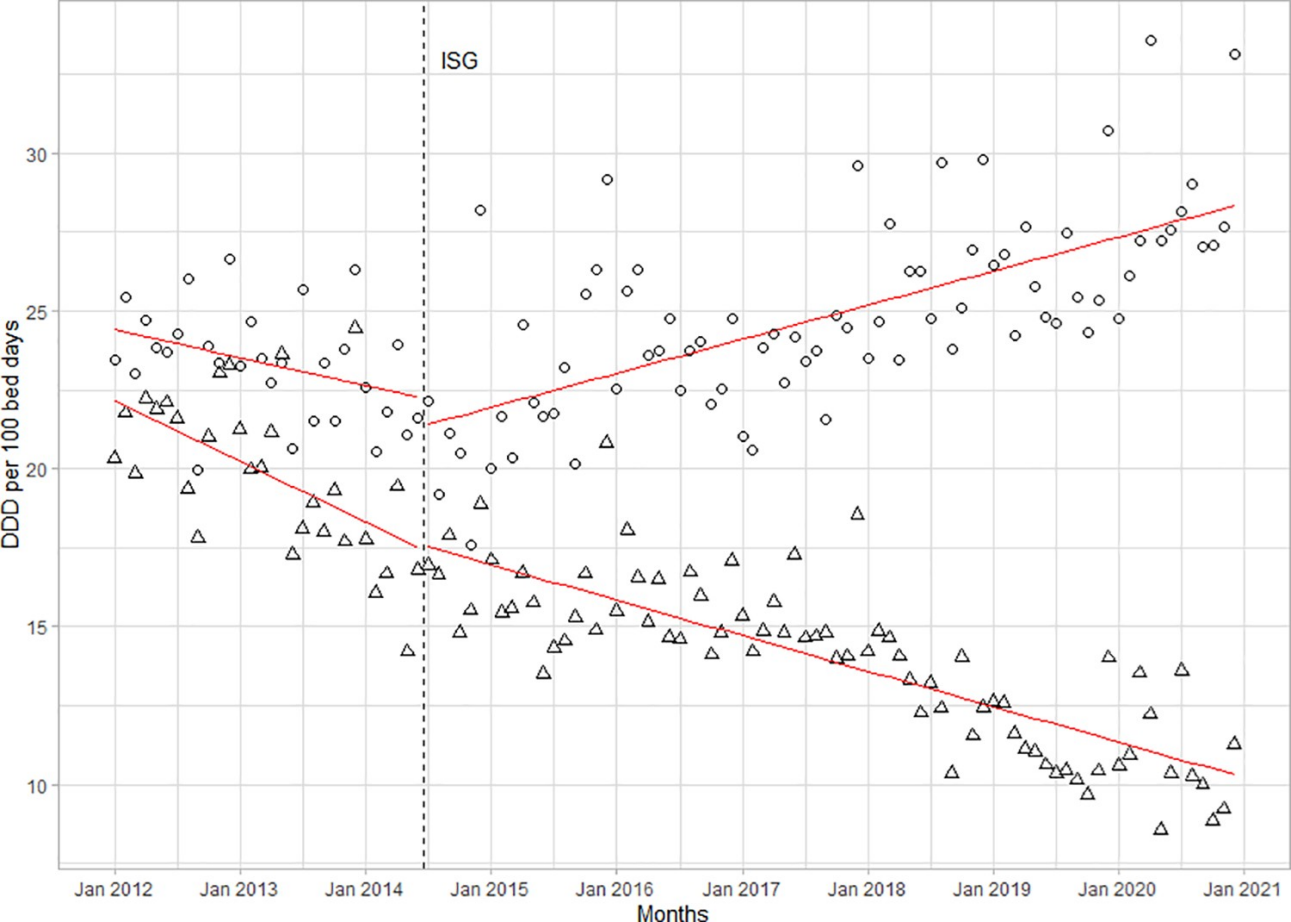
Consumption of oral and parenteral antibiotics in the Leipzig University Hospital between 2012 and 2020. Circles represent parenteral antibiotics, triangles represent oral antibiotics, and dashed line represents the introduction of institution-specific guidelines (ISGs).

The proportion of Access antibiotics increased by 2%, from 36% in 2012 to 38% in 2020. The intervention was associated with an initial reduction of -1.8 (-2.9 to -0.7, p<0.01) followed by a positive trend with a trend change of +0.13 per month (95%CI 0.08 to 0.19, p<0.001). The introduction of ISGs was associated with an increased use in parenteral antibiotics. With a trend change of +0.09 per month (95%CI 0.04 to 0.14; p<0.001), the proportion of total antibiotic consumption in DDD/100BD increased from 53% in 2012 to 72% in 2020 (**Fig 3**).

**Fig 4:**
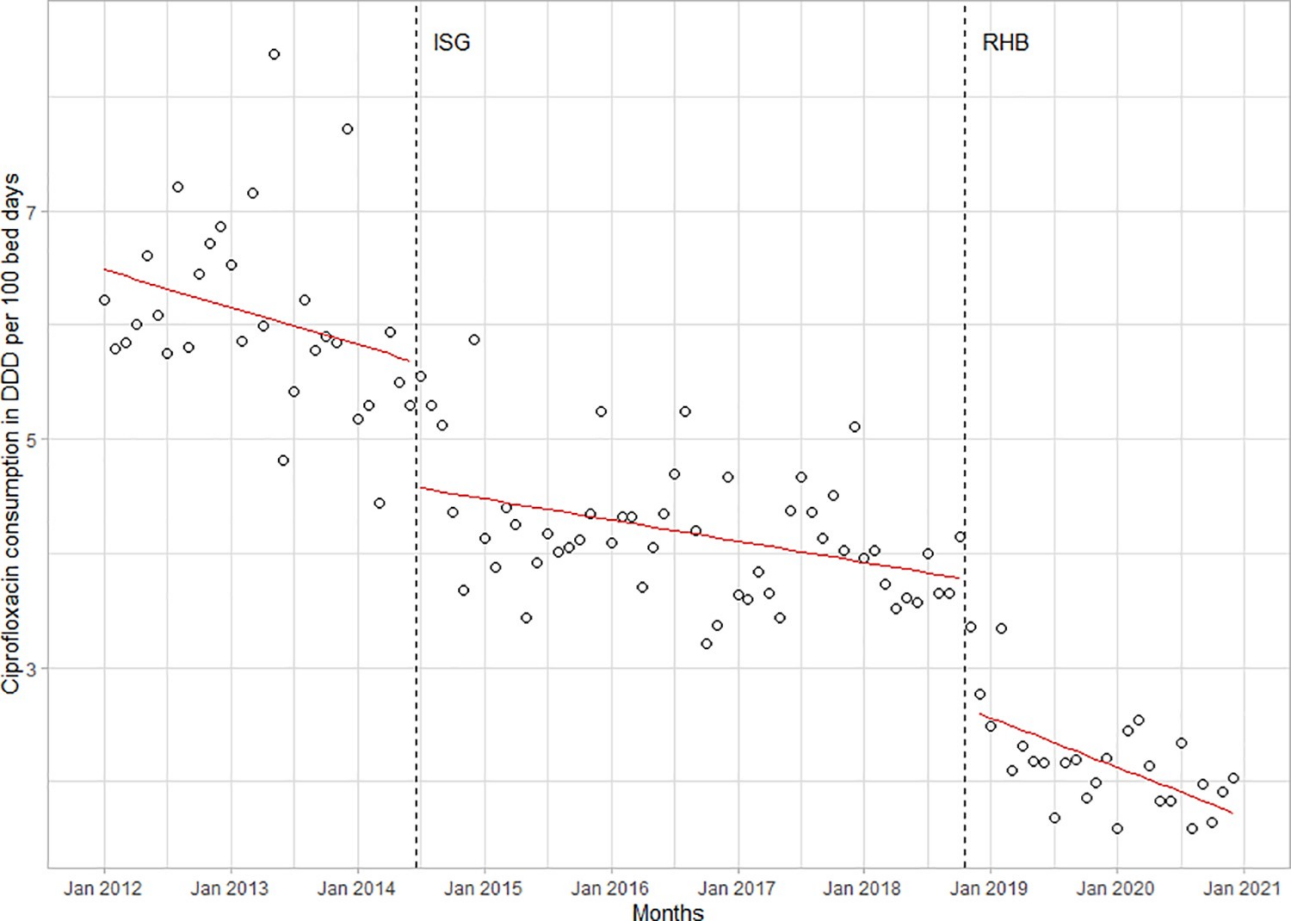
Ciprofloxacin consumption in the LUH from 2012 to 2020. Before and after both the introduction of the ISGs and the publication of warning letters (i.e., “Rote-Hand-Briefe”; RHB) by the German Federal Institute for Drugs and Medical Devices (BfArM), warning of serious adverse effects.

During the study period fluoroquinolone consumption fell by 67%, from 104 DDD/100BD in 2012 to 35 DDD/100BD in 2020. The introduction of ISGs was associated with a large, immediate reduction of ciprofloxacin consumption (-1.1 DDD/100BD; 95%CI -1.6 to -0.6; p<0.001) (**Fig 4**). A further immediate decrease affecting all fluoroquinolones occurred when the BfArM issued specific warning letters (“Rote-Hand-Briefe”) in 2018 and 2019 (-1.7 DDD/100BD, 95%CI -2.5 to -0.9, p<0.001) [32,33].

**Fig 5:**
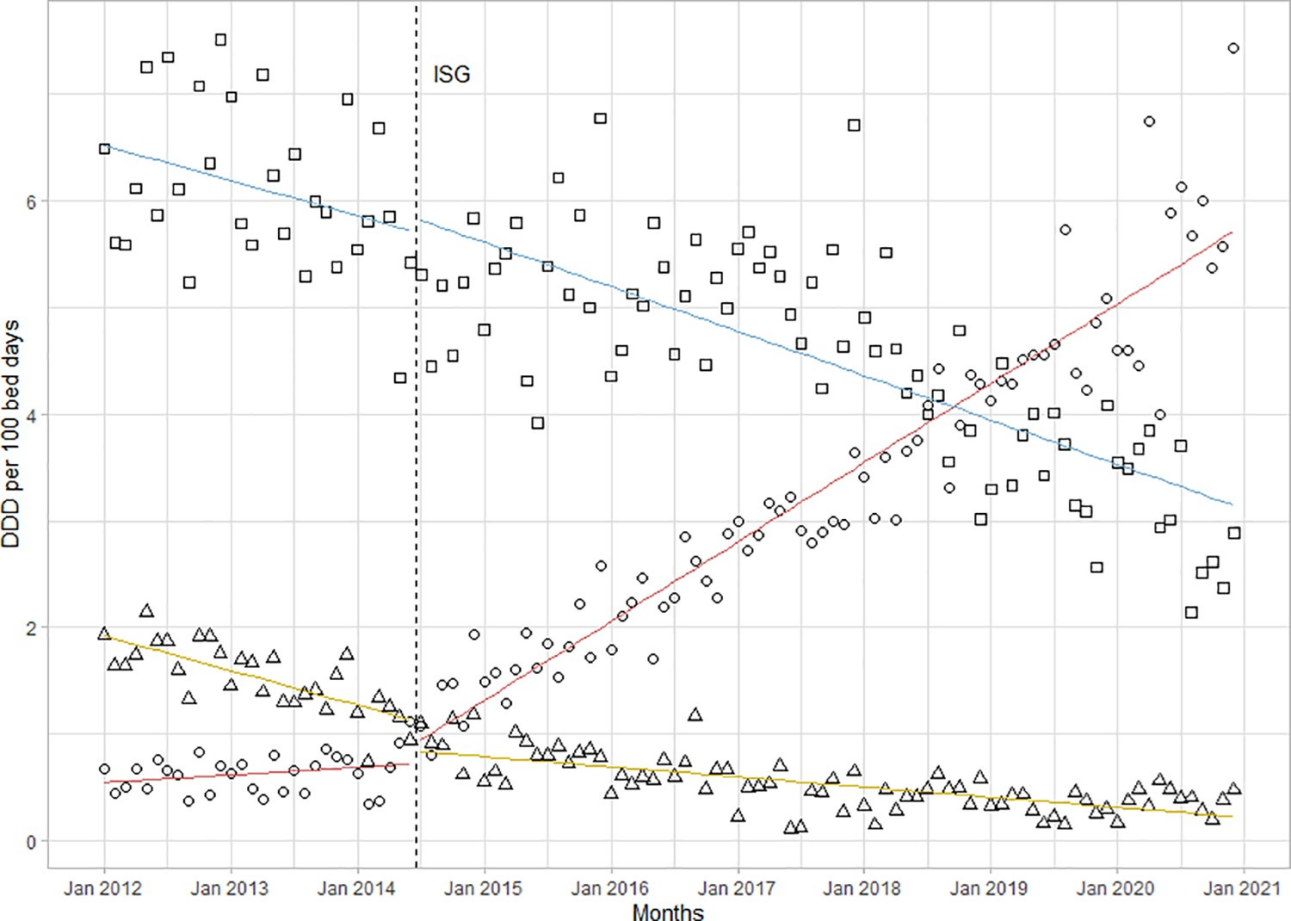
Consumption of second- and third-generation cephalosporins in the LUH from 2012 to 2020. Before and after the introduction of ISGs. Squares represent cefuroxime, triangles represent ceftriaxone, circles represent cefotaxime, and the dashed line represents the introduction of the ISGs.

For the consumption of cephalosporins, the introduction of ISGs was associated with a positive slope change of +0.08 DDD/100BD (95%CI 0.04 to 0.12, p<0.001). While overall cephalosporin consumption changed only moderately, there were major changes within the antibiotic class (**Fig 5**): cefuroxime and ceftriaxone prescriptions decreased continuously, with a significant level change for ceftriaxone of -0.3 DDD/100BD (95%CI -0.5 to -0.1, p<0.001) following the ISGs’ implementation. Conversely, for cefotaxime, the intervention was associated with a significant positive trend change of +0.06 DDD/100BD per month (95%CI 0.04 to 0.07, p<0.001).

Total penicillin consumption increased by 14% from 112 DDD/100BD in 2012 to 128 DDD/100BD in 2020, without the ISGs having a significant impact, while penicillin and beta-lactamase inhibitor combinations decreased by 14% with a significant level change of -0.96 (95%CI -1.51 to -0.42). The largest reduction (-71%) and a level change of -0.4 DDD/100BD (95%CI -0.7 to -0.1, p<0.01) was observed in sultamicillin. However, decreases were strongest in the pre-intervention phase.

**Fig 6:**
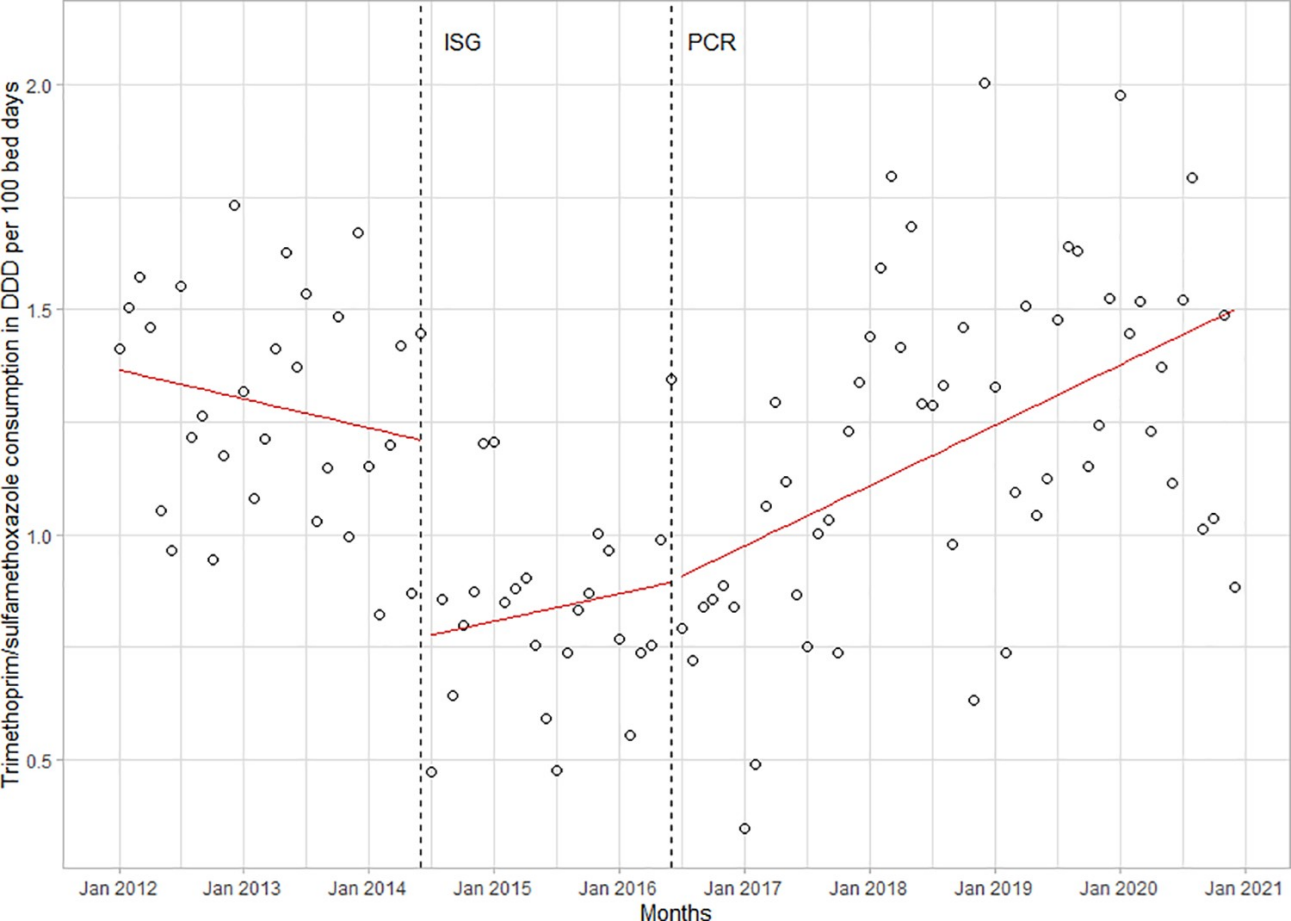
Trimethoprim/sulfamethoxazole consumption in the LUH from 2012 to 2020. Before and after both the introduction of ISGs and introduction of routine real-time polymerase chain reaction (PCR) for the diagnosis of pneumocystis pneumonia (PCP).

Trimethoprim/sulfamethoxazole prescriptions dropped immediately after the intervention by -0.4 DDD/100BD (95%CI -0.7 to -0.1, p<0.01). Prescriptions increased steadily, though not significantly, when routine *Pneumocystis jirovecii* PCR was introduced in 2016 (**Fig 6**).

Vancomycin consumption decreased pre-intervention and converted into a steady state following the intervention which was associated with a level change of +0.4 DDD/100BD (95%CI 0.1 to 0.7, p<0.01) and trend change of +0.04 DDD/100BD per month (95%CI 0.02 to 0.05, p<0.001). Additionally, we observed a level change of prescriptions of oral clarithromycin of +0.8 DDD/100BD (95%CI 0.4 to 1.1, p<0.001).

### Antibiotic consumption in wards without regular AMS rounds

Furthermore, we ran the ITSA with a subset of the data that excluded wards receiving regular AMS rounds, accounting for 51% of antibiotic consumption documented in the main dataset (**S1 Table**). Most of the changes observed in the main dataset persisted, e.g. the increase in cefotaxime consumption and a decrease in the prescription rates of fluoroquinolones, ceftriaxone, and sultamicillin. Changes in the consumption of clarithromycin and trimethoprim/sulfamethoxazole were not significant.

### Changes in resistance

**Table 3** shows annual rates of resistance to “focus antibiotics” in isolates of *K*. *pneumoniae*, *E*. *coli*, *S*. *aureus*, and *P*. *aeruginosa*.

**Table 3:** Annual antibiotic resistance rates for selected combinations of pathogens and antibiotics between 2012 and 2020 in the LUH including isolates from all microbiological samples except from routine screenings.

Pathogen	Antibiotic agent	2012 number of resistant isolates (%)	2013 number of resistant isolates (%)	2014 number of resistant isolates (%)	2015 number of resistant isolates (%)	2016 number of resistant isolates (%)	2017 number of resistant isolates (%)	2018 number of resistant isolates (%)	2019 number of resistant isolates (%)	2020 number of resistant isolates (%)	Trend: simple linear regression 2012 to 2020 (95%CI)†	Difference of percentile in 2012 vs. 2020 in %
***Escherichia coli***	Total number of isolates	3080	1656	3379	3380	3416	3262	3276	3606	3539		
	Ampicillin/sulbactam	1651 (53,6)	860 (51,9)	998 (43,8)	1476 (43,7)	1484 (43,5)	1406 (43,1)	1264 (42,6)	1381 (38,4)	1020 (28,8)	-2.4 (-3.2 to -1.5) ***	-24,8
	Piperacillin/tazobactam	267 (8,7)	204 (12,3)	142 (6,2)	214 (6,3)	189 (5,5)	204 (6,3)	153 (4,7)	128 (3,6)	103 (2,9)	-0.9 (-1.3 to -0.5) **	-5,8
	Cefotaxime	504 (16,4)	350 (21,1)	357 (15,7)	453 (13,4)	465 (13,6)	417 (12,8)	425 (13)	411 (11,4)	354 (10)	-1.0 (-1.5 to -0.5) **	-6,4
	Cefuroxime	756 (24,5)	499 (30,1)	497 (21,8)	618 (18,3)	615 (18)	567 (17,4)	557 (17)	543 (15,1)	442 (12,5)	-1.7 (-2.4 to -1.1) **	-12
	Ciprofloxacin	862 (28)	426 (25,7)	622 (27,3)	795 (23,5)	797 (23,3)	753 (23,1)	647 (19,7)	556 (15,5)	441 (12,5)	-1.8 (-2.3 to -1.3) ***	-15,5
	Trimethoprim/sulfamethoxazole	1284 (41,7)	698 (42,1)	769 (33,8)	1000 (29,6)	1028 (30,1)	930 (28,6)	836 (25,5)	942 (26,2)	979 (27,7)	-2.0 (-2.8 to -1.2) **	-14
	Imipenem	2 (0,1)	1 (0,1)	0 (0)	1 (0)	0 (0)	2 (0,1)	2 (0,1)	0 (0)	1 (0)	0.00 (-0.01 to 0.00)	-0,1
***Klebsiella pneumoniae***	Total number of isolates	902	626	537	1006	1024	782	1005	1010	995		
	Ampicillin/sulbactam	388 (43)	248 (39,6)	165 (30,7)	335 (33,3)	333 (32,6)	274 (35)	323 (35,2)	254 (25,1)	225 (22,6)	-1.9 (-2.9 to -0.9) **	-20,4
	Piperacillin/tazobactam	238 (26,4)	163 (26)	87 (16,2)	176 (17,5)	169 (16,5)	165 (21,1)	129 (12,8)	128 (12,7)	70 (7)	-2.0 (-2.9 to -1.2) **	-19,4
	Cefotaxime	219 (24,3)	128 (20,4)	87 (16,2)	153 (15,2)	157 (15,3)	129 (16,5)	165 (16,4)	135 (13,4)	146 (14,7)	-1.0 (-1.5 to -0.4) *	-9,6
	Cefuroxime	366 (40,5)	186 (29,7)	121 (22,5)	219 (21,8)	235 (23)	201 (25,7)	228 (22,7)	194 (19,2)	200 (20,1)	-1.8 (-3.0 to -0.6) *	-20,4
	Ciprofloxacin	254 (28,2)	130 (20,8)	100 (18,6)	190 (18,9)	201 (19,6)	166 (21,2)	186 (18,5)	110 (10,9)	138 (13,9)	-1.4 (-2.2 to -0.6) **	-14,3
	Trimethoprim/sulfamethoxazole	294 (32,6)	143 (22,8)	130 (24,2)	201 (20)	231 (22,6)	181 (23,2)	211 (21)	164 (16,2)	199 (20)	-1.2 (-2.0 to -0.4) *	-12,6
	Imipenem	34 (3,8)	11 (1,8)	0 (0)	12 (1,2)	8 (0,8)	2 (0,3)	3 (0,3)	4 (0,4)	0 (0)	-0.3 (-0.5 to -0.1) *	-3,8
***Pseudomonas aeruginosa***	Total number of isolates	826	646	365	973	899	864	788	931	940		
	Piperacillin/tazobactam	212 (25,7)	137 (21,2)	53 (14,5)	124 (12,8)	115 (12,8)	137 (15,9)	118 (15)	96 (10,3)	84 (8,9)	-1.6 (-2.4 to -0.8) **	-16,8
	Ceftazidime	207 (25,1)	142 (22)	40 (11)	119 (12,2)	137 (15,2)	139 (16,1)	112 (14,2)	89 (9,6)	95 (10,1)	-1.4 (-2.4 to -0.5) *	-15
	Ciprofloxacin	202 (24,5)	183 (28,3)	92 (25,2)	240 (24,7)	211 (23,5)	227 (26,3)	139 (17,6)	124 (13,3)	117 (12,4)	-1.8 (-2.6 to -0.9) **	-12,1
	Meropenem	65 (7,9)	54 (8,4)	16 (5)	50 (5,1)	67 (7,4)	39 (4,5)	40 (5,1)	41 (4,4)	30 (3,2)	-0.5 (-0.8 to -0.2) *	-4,7
	Imipenem	140 (16,9)	136 (21,1)	76 (20,8)	257 (26,4)	239 (26,5)	208 (24,1)	146 (18,5)	135 (14,5)	129 (13,7)	-0.7 (-1.9 to 0.5)	-3,2
***Staphylococcus aureus***	Total number of isolates	1719	1741	1103	2339	2468	2268	2234	2317	2236		
	Penicillin G	1193 (69,3)	1199 (68,9)	847 (76,9)	1360 (58,1)	1471 (59,7)	1358 (59,9)	1303 (58,4)	1279 (55,1)	1213 (54,3)	-2.3 (-3.5 to -1.1) **	-15
	Oxacillin	291 (16,9)	320 (18,4)	496 (45)	164 (7)	193 (7,8)	170 (7,5)	134 (6)	142 (6,1)	105 (4,7)	-2.7 (-5.6 to 0.1)	-12,2
	Ciprofloxacin	514 (29,9)	516 (29,6)	566 (51,3)	458 (19,6)	491 (19,9)	442 (19,5)	381 (17,1)	366 (15,8)	316 (14,1)	-2.9 (-5.2 to -0.6) *	-15,8
	Clindamycin	193 (11,2)	190 (10,9)	216 (19,6)	112 (4,8)	141 (5,7)	120 (5,3)	99 (4,4)	88 (3,8)	83 (3,7)	-1.4 (-2.4 to -0.3) *	-7,5
	Levofloxacin	490 (28,6)	479 (27,5)	551 (50)	428 (18,3)	465 (18,9)	414 (18,2)	352 (15,8)	345 (14,9)	291 (13)	-2.8 (-5.1 to -0.5) *	-15,6
	Moxifloxacin	497 (28,9)	489 (28,1)	554 (50,3)	428 (18,3)	465 (18,9)	416 (18,3)	357 (16)	343 (14,8)	310 (13,9)	-2.8 (-5.1 to -0.5) *	-15
	Roxithromycin	347 (20,2)	334 (19,2)	284 (25,7)	309 (13,2)	388 (15,7)	399 (17,6)	355 (15,9)	403 (17,3)	361 (16,2)	-0.6 (-1.5 to 0.2)	-4
	Trimethoprim/sulfamethoxazole	316 (18,5)	350 (20,1)	69 (6,3)	154 (6,6)	191 (7,8)	150 (6,7)	49 (2,2)	10 (0,4)	150 (6,7)	-1.9 (-3.0 to -0.8) *	-11,8
	Vancomycin	2 (0,1)	1 (0,1)	2 (0,2)	4 (0,2)	1 (0)	0 (0)	1 (0)	1 (0)	1 (0)	-0.01 (-0.03 to 0.00)	-0,1

* = p < 0.05

** = p < 0.01

*** = p < 0.001.

More comprehensive tables including further antibiotic agents as well as *E*. *faecium*, *E*. *faecalis*, and *S*. *epidermidis* isolates can be found in **S2 Table**, and ITSA results of resistance rates can be found in **S3 Table**. Rates of resistance to the majority of tested antibiotics, including broad-spectrum penicillins, second and third generation cephalosporins, fluoroquinolones and trimethoprim/sulfamethoxazole decreased significantly in all analyzed pathogens, except in enterococci, where no significant trends were observed. In most cases, resistance rates were already decreasing in the pre-intervention phase and continued to decrease with a more moderate trend after the intervention. Negative trend changes were observed for ciprofloxacin resistance in *E*. *coli*, *P*. *aeruginosa* and *S*. *aureus* as well as cefotaxime resistance in *E*. *coli*, but these changes were not significant.

Fluoroquinolone-resistance rates among *Enterobacterales*, staphylococci, and *P*. *aeruginosa* decreased strongly, with relative reductions of ciprofloxacin-resistance ranging between 12% in *P*. *aeruginosa* (-1.8% per year, 95%CI -2.6 to -0.9, p<0.01) to 16% in *S*. *aureus* (-2.9% per year; 95%CI -5.2 to -0.6, p<0.05).

Resistance to second- and third-generation cephalosporins, tested with cefuroxime and cefotaxime, decreased significantly in *Enterobacterales*. For instance, rates of cefuroxime-resistant *K*. *pneumoniae* decreased by 1.8% per year (95%CI -3.0 to -0.6, p<0.05) from 40.5% (366 of 902 cases) in 2012 to 20.1% (200 of 996 cases) in 2020. Similarly, we observed reduced rates of ceftazidime resistance in *P*. *aeruginosa* (-1.4% per year; 95%CI -2.4 to -0.5; p<0.05).

We observed reduced rates of resistance to broad-spectrum penicillins among *Enterobacterales*, as well as staphylococci and pseudomonas. Ampicillin/sulbactam-resistant *E*. *coli* decreased significantly by 2.4% per year (95%CI -3.2 to -1.5; p<0.001) accounting for a relative reduction of almost 25% over the study period. A comparable trend of -1.9 (95%CI -2.9 to -0.9, p<0.01) was observed for ampicillin/sulbactam-resistant *K*. *pneumoniae*. In staphylococci, significantly decreased rates of oxacillin-resistance are indicative of reduced resistance rates to other broad-spectrum penicillins. Interestingly, rates of penicillin G resistance in *S*. *aureus*, which is expected in penicillinase-producing strains, showed a significant decreasing trend of -2.3% per year (95%CI -3.5 to -1.1; p<0.01), accounting for a relative reduction of 15% from 2012 to 2020. Rates of piperacillin/tazobactam resistance decreased significantly, resulting in a relative reduction of almost 20% in *K*. *pneumoniae* (-2% per year; 95%CI -2.9 to -1.2; p<0.01), and 17% in *P*. *aeruginosa*. (-1.6% per year; 95%CI -2.4 to -0.8; p<0.01).

Trimethoprim/sulfamethoxazole resistance showed an overall significant decreasing trend in *Enterobacterales* and staphylococci (e.g. -1.7% per year [95%CI -2.0 to -1.4, p<0.001] in *E*. *coli*, and -2.9% per year [95%CI -4.0 to -1.7, p<0.001] in *S*. *epidermidis*), yet in all of them an increase could be observed in 2020.

Vancomycin-resistance in staphylococci stayed at an extremely low level (0–0.2%).

### Comparison with data from EARS-Net

**Fig 7:**
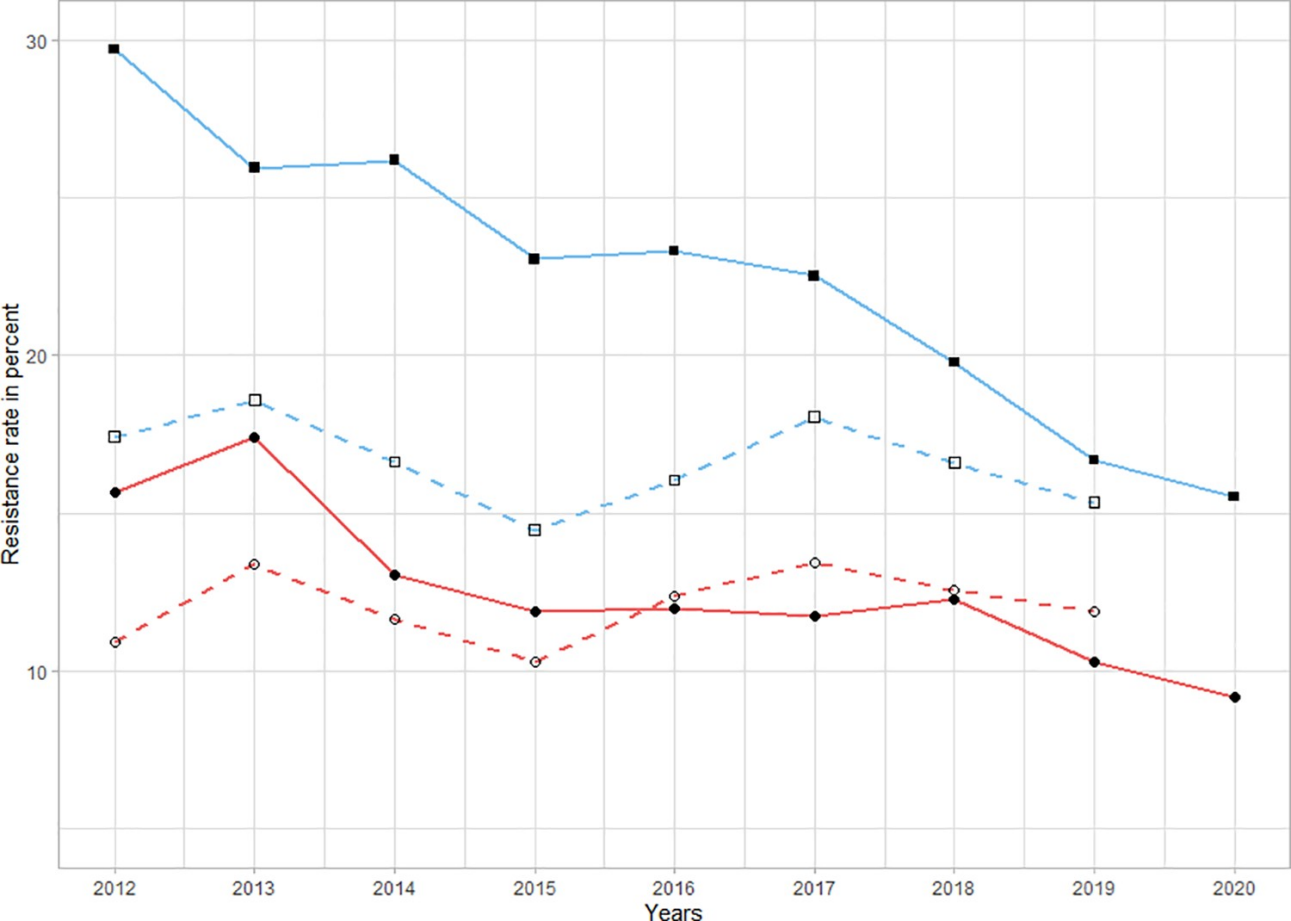
Resistance rates to fluoroquinolones and third-generation cephalosporins in selected *Enterobacterales* (*Klebsiella pneumoniae* and *Escherichia coli*) in the LUH between 2012 and 2020. Plotted annually, compared with the German average reported in the European Antimicrobial Resistance Surveillance Network (EARS-Net) between 2012 and 2020. Blue lines and squares represent rates of fluoroquinolone resistance. Red lines and circles represent third-generation cephalosporin resistance rates. Full lines and shapes represent resistance rates in the LUH, dashed lines and empty shapes represent German resistance rates.

**S4 Table** shows annual resistance rates of *E*. *coli*, *K*. *pneumoniae*, *P*. *aeruginosa*, and *S*. *aureus* in Germany between 2012 and 2019 [26]. Methicillin-resistant *S*. *aureus* (MRSA) rates decreased by 1.2% per year (95%CI -1.3 to -1.0, p<0.001), while decreases in other pathogens were very moderate. In the case of third-generation cephalosporin-resistant *E*. *coli*, rates actually increased (0.38%; 95%CI 0.18 to 0.58, p<0.001). A graphic comparison of resistance rates to fluoroquinolones and third-generation cephalosporins in *Enterobacterales* is given in **Fig 7**.

### CDI epidemiology

We observed a large reduction of CDI cases. In 2014, when the ISGs were introduced, culture positive cases had declined by 47% when compared with the previous year (279 vs. 523 cases). Over the whole study period annual CDIs were reduced by 65%, from 501 in 2012 to 174 in 2020 (**Table 4**).

**Table 4:** Annual culture-positive *Clostridioides difficile* cases in the LUH between 2012 and 2020.

Year	2012	2013	2014	2015	2016	2017	2018	2019	2020	Trend: simple linear regression 2012 to 2020 (95%CI)	Difference 2012 to 2020 in %
**Culture-positive *Clostridioides difficile* cases**	501	523	279	290	265	207	168	169	174	-44.6 (61.2 to 28.0) **	-65%

## Discussion

In this study, we observed significant reductions in antibiotic use and antibiotic resistance. Most of these occurred in the pre-intervention phase, likely due to other AMS interventions implemented in 2012. Prescription reductions are expected to be greatest in the first few years after the introduction of AMS interventions and to stabilize after a few years. Since the ISGs were implemented after large initial reductions had already been achieved, the naturally more moderate decreases in antibiotic use and resistance a few years after the start of the AMS program appear as positive trend changes in the ITSA of this intervention. Therefore, we do not consider the positive trend changes in total antibiotic consumption as well as in sultamicillin, ceftriaxone and fluoroquinolones to be a result of the intervention. The same applies for the ITSA of resistance rates.

Overall, it appears that the ISGs did not have a relevant impact on the total quantity of antibiotic prescriptions. However, despite the challenges in interpreting the data, we observed several changes in antibiotic consumption that were clearly associated with the intervention and in line with our predictions, including decreased prescriptions of ciprofloxacin and trimethoprim/sulfamethoxazole, and increased use of cefotaxime and clarithromycin. The ISGs seem to have further contributed to reductions in sultamicillin and ceftriaxone. However, in these cases the result of the ITSA is less reliable given the steep decrease in the pre-intervention phase. The increased trend and level of vancomycin use can likely be explained with the recommendation for the treatment of CDIs. The ISGs recommend high doses (500 mg QID) of orally-administered vancomycin whereas lower doses (125–250 mg QID) were used before 2014.

Major changes in antibiotic consumption related to the implementation of ISGs, which were found in the analysis of the data from all departments involved, persisted in the analysis of antibiotic use in a subgroup that did not include wards with regular visits by the AMS team (**S1 Table**). We therefore believe that the ISGs had a relevant independent effect on antibiotic prescriptions. A major unintended effect associated with the ISGs’ implementation was the significant positive-trend change of parenteral applications. This was, in part, likely a result of the successful efforts to reduce the use of sultamicillin, cefuroxime, and ciprofloxacin for empirical antimicrobial treatment, as well as the frequent recommendation of cefotaxime and other parenterally administered antibiotics. A strong potential confounder that may have contributed to this trend is the fact that the German hospital financing system is under increasing pressure to reduce the length of hospital stays. In many cases it is considered necessary that patients are discharged from the hospital as soon as oral administration of antibiotics is possible to avoid financial sanctions. Nevertheless, this finding suggests that the goal of sequential parenteral to oral therapy might not be sufficiently met, which would be in line with responses in a previous survey-based study where several respondents pointed out the need for recommendations for oral antimicrobial therapy [17].

Over the study period, we observed large decreases in antibiotic resistance in almost all key pathogens and for several frequently-used antibiotic groups. Despite many limitations it seems likely that the reductions in resistance are a result of the decreases and changes in antibiotic consumption. It is important to note that the baseline resistance rates in Germany were much lower than that for LUH (as a result a lesser decline would be expected for Germany overall), and that 2019 resistance rates for Germany and LUH were similar [26]. The decreasing trend in oxacillin-resistant *S*. *aureus* could be the product of a similar national trend of declining MRSA rates. Yet, this seems not to be the case in other pathogens, particularly for resistance to fluoroquinolones and cephalosporins, for which the national reference resistance rates decreased at most subtly. The quality of the resistance data did not allow for a conclusive analysis of the ISGs’ effect on resistance rates. Given the large reductions in antibiotic use and resistance achieved prior to their introduction, their impact is likely to have been small compared to previous interventions. However, it seems probable that the changes in the use of ciprofloxacin, cephalosporins, and ampicillin/sulbactam associated with the ISGs’ introduction did contribute to the respective decreases in resistance observed in the post-intervention phase.

One finding of particular interest was that the increased use of third-generation cephalosporins was associated with a significant decrease in resistance to third-generation cephalosporins in *E*. *coli* and *K*. *pneumoniae*. This is especially noteworthy as the national trend of third-generation cephalosporin-resistant *E*. *coli* increased significantly over the study period. We attribute this to the switch from ceftriaxone to cefotaxime within the group of third-generation cephalosporins. One explanation could be that ceftriaxone, as a biliary-excreted antibiotic, might be a stronger driver for resistance in *Enterobacterales* than cefotaxime [35–37]. The switch from ceftriaxone to cefotaxime might have also contributed to the drastic reduction of CDI (-65%), as proposed in a previous study [38]. Typically, third-generation cephalosporins are considered high-risk antibiotics for the development of CDIs [39]. The fact that CDI declined dramatically despite an overall increase in the use of third-generation cephalosporins suggests that the specific antibiotic agent matters in this case. Additionally, we believe that reduced use of other high-risk antibiotics such as fluoroquinolones likely contributed to further reductions in CDIs [39,40]. The largest annual reduction (47%) was observed in the year when ISGs were implemented, from 523 cases in 2013 to 279 cases in 2014. It is unlikely that this reduction was mainly due to the ISGs as they were only in place in the second half of 2014, although due to their impact on the high-risk antibiotics mentioned above, they likely contributed to further reductions in subsequent years.

### Limitations

Since this study is based on routinely documented data without a control group, we can only assess associations and not causations. To analyze antibiotic consumption, we used the pharmacy records of the dispensed antibiotics. Dispensing data holds several limitations. Most importantly, it does not include information on when and whether the dispensed antibiotics were actually used and whether the prescriptions were indicated. Complete surveillance for antibiotic consumption and resistance was only available from the year 2012, when most AMS interventions started. It was therefore not possible to analyze changes due to previous AMS interventions using ITSA.

The fact that antibiotic consumption and resistance rates were already strongly decreasing before the implementation of ISGs makes analyzing their impact with ITSA challenging. We regard other AMS interventions as the strongest confounder. Aside from these AMS interventions, there are many other potential confounders affecting antibiotic consumption, such as new standard operating procedures (SOP) and other department-specific recommendations, as well as stockouts or changed antibiotic prices. Additionally, the current COVID-19 pandemic likely impacted prescriptions in 2020. We addressed such factors wherever possible: by adding a second intervention in the case of fluoroquinolones and trimethoprim/sulfamethoxazole, removing data points of ampicillin/sulbactam affected by a stockout, and analyzing consumption data again after excluding wards receiving AMS rounds.

Resistance rates and CDI numbers were only available as annual aggregates. Hence, a clearly separated microanalysis of resistance rates stratified by those wards receiving regular AMS rounds and those not (especially “heavv users” such as hematologic oncology wards) using aggregated data, is not feasible. The small number of data points and the strong pre-intervention trend make ITSA unreliable for this data. It is therefore not possible to draw clear conclusions regarding the effect of the ISGs on resistance rates and CDIs. Aside of the antimicrobial treatment, there are several confounders that might have impacted resistance rates in our hospital. For example, infection prevention and control interventions may have reduced hospital-acquired infections and thus contributed to lower incidences of CDIs and antibiotic resistance. In addition, given the various factors contributing to the rise and spread of AMR, it is expected that rates of resistance will be largely influenced by external factors, including treatments from other providers. To address this, we compared the resistance trends in our hospital with national trends.

## Conclusions

This hospital-wide study demonstrated that well-implemented ISGs can have a significant immediate and lasting impact on the choice of agents for antimicrobial therapy. At our institution, ISGs appeared to have contributed to large reductions in resistance rates and incidences of CDI. Hence, we recommend the wider use of ISGs for empirical antibiotic therapy because they are relatively easy to implement, reach all clinicians, and have a significant impact on antibiotic prescribing. We believe that our findings are generalisable to hospitals with a supportive AMS environment and a sufficient awareness of AMR among prescribers.

## Supporting information

S1 Fig Number of monthly bed days (BD) in the Leipzig University Hospital between 2012 and 2020. Triangles represent BD in the old documentation, circles represent bed days in the new documentation, and the solid line represents adjusted BD used for the calculation of DDD/100BD.
(TIF)

S1 Table ITSA of changes in dispensed antimicrobials, excluding wards served by the AMS team–subset. (DOCX)

S2 Table Annual resistance rates for selected combinations of pathogens and antibiotic agents between 2012 and 2020 in the Leipzig University Hospital–full dataset. (DOCX)

S3 Table ITSA results of changes in resistance rates in the Leipzig University Hospital between 2012 and 2020, associated with the introduction of ISGs. (DOCX)

S4 Table Annual German reference antibiotic resistance rates and trends for selected combinations of pathogens and antibiotic agents between 2012 and 2019, collected from the European Antimicrobial Resistance Network (EARS-Net). (DOCX)
