# Supplementary figures and images for: Changes in antibiotic consumption, AMR and Clostridioides difficile infections in a large tertiary-care center following the implementation of institution-specific guidelines for antimicrobial therapy: A nine-year interrupted time series study
Source: PLoS One. 2021 Oct 14;16(10):e0258690. doi: 10.1371/journal.pone.0258690 (PMC8516227; doi:10.1371/journal.pone.0258690)

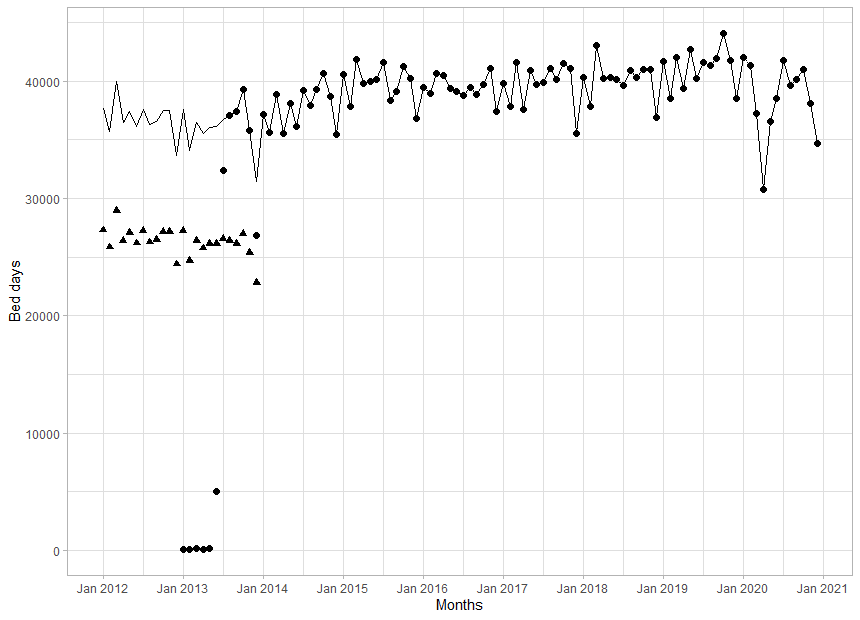

Supplement: S1 Fig — Triangles represent BD in the old documentation, circles represent bed days in the new documentation, and the solid line represents adjusted BD used for the calculation of DDD/100BD. (TIF) [file pone.0258690.s001.tif]
