# Supplementary material for: Changes in antibiotic consumption, AMR and Clostridioides difficile infections in a large tertiary-care center following the implementation of institution-specific guidelines for antimicrobial therapy: A nine-year interrupted time series study
Source: PLoS One. 2021 Oct 14;16(10):e0258690. doi: 10.1371/journal.pone.0258690 (PMC8516227; doi:10.1371/journal.pone.0258690)
Supplement: S1 Table — (DOCX) [file pone.0258690.s002.docx]

**S1 Table.** Results of the interrupted time series analysis of changes in dispensation of antimicrobials in a subset of the total antibiotic consumption in the LUH that did not include wards with regular AMS rounds (i.e. intensive care units, pneumology, gastroenterology, and two surgical wards) as well as hematologic oncology wards. As in the main dataset, psychiatric units, pediatric units and outpatient departments were excluded from the analysis. The study period was from 2012 to 2020. The first intervention was the introduction of ISGs. Antibiotic-specific interventions were, for fluoroquinolones: two warning letters [“Rote Hand Briefe”] issued by the German Federal Institute for Drugs and Medical Devices (BfArM) in October 2018 and April 2019 warning of serious adverse effects of fluoroquinolones; for trimethoprim/sulfamethoxazole: the introduction of routine real-time PCR for the diagnosis of pneumocystis pneumonia [PCP]).

| **Antibiotics (application)** | **Baseline antibiotic consumption in RDD/100BD (β0)** | **Baseline trend (increase in RDD/100BD per month) (β1)** | **Level change 1 month after the intervention in RDD/100BD per month (β2)** | **Trend change after the intervention in RDD/100BD (β3)** | **Level change 1 month after the 2nd intervention in RDD/100BD per month (β4)** | **Trend change after the 2nd intervention in RDD/100BD (β5)** |
| --- | --- | --- | --- | --- | --- | --- |
| **Total antibiotic consumption** |  |  |  |  |  |  |
| Antibiotics | 25.7 (24.3 to 27.1) *** | -0.14 (-0.22 to -0.06) ** | -1.2 (-2.8 to 0.4) | 0.15 (0.07 to 0.23) *** |  |  |
| Antibiotics (P) | 12.2 (11.3 to 13) *** | -0.03 (-0.08 to 0.02) | -1.1 (-2 to -0.1) * | 0.09 (0.04 to 0.14) *** |  |  |
| Antibiotics (O) | 13.5 (12.6 to 14.4) *** | -0.11 (-0.16 to -0.06) *** | -0.1 (-1.1 to 0.9) | 0.06 (0.01 to 0.11) * |  |  |
| **WHO AWaRe Classification** |  |  |  |  |  |  |
| Access | 11.2 (10.5 to 12) *** | -0.07 (-0.11 to -0.02) ** | -1.3 (-2.1 to -0.4) ** | 0.09 (0.04 to 0.13) *** |  |  |
| Watch | 15 (14.1 to 15.9) *** | -0.09 (-0.14 to -0.04) ** | -0.3 (-1.3 to 0.8) | 0.08 (0.03 to 0.14) ** |  |  |
| Reserve | 0.2 (0 to 0.3) ** | 0 (0 to 0.01) | 0 (-0.2 to 0.1) | 0 (0 to 0.01) |  |  |
| **Penicillins** | 6.9 (6.2 to 7.5) *** | -0.02 (-0.06 to 0.02) | -0.5 (-1.2 to 0.2) | 0.03 (-0.01 to 0.07) |  |  |
| Ampicillin/sulbactam (P) | 1.6 (1.4 to 1.8) *** | 0 (-0.01 to 0.02) | -0.4 (-0.6 to -0.2) *** | 0 (-0.01 to 0.01) |  |  |
| Sultamicillin (O) | 2.4 (2.2 to 2.5) *** | -0.03 (-0.04 to -0.02) *** | -0.4 (-0.6 to -0.2) *** | 0.02 (0.01 to 0.03) *** |  |  |
| Piperacillin/tazobactam (P) | 0.9 (0.8 to 1) *** | 0 (-0.01 to 0.01) | 0 (-0.1 to 0.1) | 0 (-0.01 to 0.01) |  |  |
| **Cephalosporins** | 7.7 (7.2 to 8.2) *** | -0.04 (-0.07 to -0.01) ** | -0.4 (-1 to 0.2) | 0.05 (0.02 to 0.08) ** |  |  |
| 2G Cephalosporins | 5.7 (5.2 to 6.1) *** | -0.03 (-0.05 to 0) * | 0.1 (-0.4 to 0.6) | 0 (-0.03 to 0.02) |  |  |
| 3G Cephalosporins | 1.6 (1.3 to 1.9) *** | -0.01 (-0.02 to 0.01) | -0.5 (-0.8 to -0.1) ** | 0.03 (0.02 to 0.05) *** |  |  |
| Cefotaxime (P) | 0.4 (0.2 to 0.6) *** | 0 (-0.01 to 0.01) | -0.1 (-0.3 to 0.1) | 0.04 (0.03 to 0.05) *** |  |  |
| Ceftriaxone (P) | 0.9 (0.8 to 1) *** | -0.01 (-0.02 to -0.01) *** | -0.2 (-0.3 to -0.1) ** | 0.01 (0 to 0.01) * |  |  |
| Cefuroxime (O) | 3.4 (3 to 3.7) *** | -0.02 (-0.04 to 0) | 0 (-0.4 to 0.4) | -0.01 (-0.03 to 0.01) |  |  |
| Cefuroxime (P) | 2.3 (2.1 to 2.5) *** | -0.01 (-0.02 to 0) | 0.1 (-0.1 to 0.3) | 0.01 (0 to 0.02) |  |  |
| **Fluoroquinolones** | 3.9 (3.5 to 4.3) *** | -0.03 (-0.05 to -0.01) ** | -0.2 (-0.7 to 0.3) | 0.03 (0 to 0.05) * | -0.7 (-1.2 to -0.2) ** | -0.02 (-0.05 to 0.01) |
| Ciprofloxacin | 2.6 (2.3 to 2.9) *** | -0.01 (-0.03 to 0) | -0.5 (-0.8 to -0.2) ** | 0.01 (-0.01 to 0.03) | -0.3 (-0.7 to 0) | -0.02 (-0.04 to 0.003) |
| Moxifloxacin | 0.6 (0.5 to 0.8) *** | 0 (-0.01 to 0) | 0.1 (-0.1 to 0.2) | 0 (-0.01 to 0.01) | -0.05 (-0.2 to 0.1) | 0.003 (-0.007 to 0.01) |
| Levofloxacin | 0.7 (0.5 to 0.9) *** | -0.01 (-0.02 to 0) * | 0.2 (0 to 0.5) * | 0.02 (0.01 to 0.03) ** | -0.3 (-0.6 to -0.1) ** | -0.006 (-0.02 to 0.01) |
| **Carbapenems** | 1.1 (1 to 1.2) *** | -0.01 (-0.02 to 0) * | -0.2 (-0.3 to 0) * | 0.01 (0 to 0.02) ** |  |  |
| Meropenem (P) | 0.3 (0.2 to 0.4) *** | 0 (-0.01 to 0) | 0 (-0.1 to 0.1) | 0 (0 to 0.01) * |  |  |
| Imipenem (P) | 0.8 (0.7 to 0.9) *** | -0.01 (-0.01 to 0) * | -0.1 (-0.2 to 0) * | 0.01 (0 to 0.01) |  |  |
| **Other antibiotic groups** |  |  |  |  |  |  |
| Trimethoprim/sulfamethoxazole | 0.8 (0.7 to 0.9) *** | -0.01 (-0.02 to 0) ** | -0.1 (-0.3 to 0) | 0.01 (0 to 0.02) ** |  |  |
| Clarithromycin (O) | 0.7 (0.6 to 0.9) *** | 0 (-0.02 to 0.01) | 0.2 (0 to 0.4) | 0 (-0.01 to 0.01) |  |  |
| Clindamycin | 1.5 (1.2 to 1.7) *** | 0.01 (-0.01 to 0.02) | 0 (-0.3 to 0.3) | 0 (-0.01 to 0.02) |  |  |
| Vancomycin | 0.7 (0.6 to 0.8) *** | -0.01 (-0.01 to 0) * | 0 (-0.2 to 0.1) | 0.01 (0.01 to 0.02) *** |  |  |
| Metronidazole (P) | 0.8 (0.7 to 0.9) *** | -0.01 (-0.01 to 0) * | -0.1 (-0.2 to 0) ** | 0.01 (0 to 0.01) * |  |  |
| Metronidazole (O) | 0.5 (0.5 to 0.6) *** | 0 (-0.01 to 0) ** | -0.1 (-0.2 to -0.1) *** | 0 (0 to 0) |  |  |

**Abbreviations:** (P) = parenteral, (O) = oral. Legend: * = p < 0.05; ** = p < 0.01; *** = p < 0.001
