# Supplementary material for: Changes in antibiotic consumption, AMR and Clostridioides difficile infections in a large tertiary-care center following the implementation of institution-specific guidelines for antimicrobial therapy: A nine-year interrupted time series study
Source: PLoS One. 2021 Oct 14;16(10):e0258690. doi: 10.1371/journal.pone.0258690 (PMC8516227; doi:10.1371/journal.pone.0258690)
Supplement: S2 Table — (DOCX) [file pone.0258690.s003.docx]

**S2 Table.** Annual antibiotic resistance rates for selected combinations of pathogens and antibiotic agents between 2012 and 2020 in the LUH, consisting of isolates from all microbiological samples except routine screenings.

| **Pathogen** | **Substance** | **2012 number of resistant isolates (%)** | **2013 number of resistant isolates (%)** | **2014 number of resistant isolates (%)** | **2015 number of resistant isolates (%)** | **2016 number of resistant isolates (%)** | **2017 number of resistant isolates (%)** | **2018 number of resistant isolates (%)** | **2019 number of resistant isolates (%)** | **2020 number of resistant isolates (%)** | **Trend (simple linear regression)** | **Relative change of resistance-rates between 2012 and 2020 in %** |
| --- | --- | --- | --- | --- | --- | --- | --- | --- | --- | --- | --- | --- |
| ***Acinetobacter baumannii*** | **Total number of Isolates** | **197** | **179** | **58** | **166** | **189** | **186** | **158** | **63** | **46** |  |  |
|  | Ciprofloxacin | 33 (16,8) | 25 (14) | 3 (5,2) | 11 (6,6) | 15 (7,9) | 16 (8,6) | 16 (10,1) | 2 (3,1) | 9 (19,6) | -0.2 (-1.6 to 1.3) | +2,8 |
|  | Levofloxacin | 30 (15,2) | 21 (11,7) | 3 (5,2) | 11 (6,6) | 14 (7,4) | 15 (8,1) | 14 (8,9) | 1 (1,6) | 9 (19,6) | -0.1 (-1.5 to 1.4) | +4,4 |
|  | Imipenem | 3 (1,5) | 7 (3,9) | 1 (1,7) | 8 (4,8) | 8 (4,2) | 1 (0,5) | 12 (7,5) | 1 (1,6) | 7 (15,2) | +0.9 (-0.1 to 1.9) | +13,7 |
|  | Meropenem | 2 (1) | 4 (2,2) | 0 (0) | 7 (4,2) | 8 (4,2) | 1 (0,5) | 11 (6,9) | 1 (1,6) | 7 (15,2) | +1.1 (0.1 to 2.1) | +14,2 |
|  | Trimethoprim/  sulfamethoxazole | 10 (5,1) | 8 (4,5) | 0 (0) | 10 (6) | 5 (2,6) | 3 (1,6) | 9 (5,7) | 0 (0) | 2 (4,3) | -0.2 (-0.8 to 0.5) | -0.8 |
| ***E. coli*** | **Total number of Isolates** | **3080** | **1656** | **2278** | **3380** | **3416** | **3262** | **3276** | **3606** | **3539** |  |  |
|  | Ampicillin/sulbactam | 1651 (53,6) | 860 (51,9) | 998 (43,8) | 1476 (43,7) | 1484 (43,5) | 1406 (43,1) | 1264 (42,6) | 1381 (38,4) | 1020 (28,8) | -2.4 (-3.2 to -1.5) *** | -24,8 |
|  | Ampicillin | 1894 (61,5) | 1017 (61,5) | 1236 (54,3) | 1812 (53,7) | 1797 (52,7) | 1717 (52,7) | 1797 (54,9) | 1833 (50,8) | 1706 (48,2) | -1.4 (-2.0 to -0.8) ** | -13,3 |
|  | Piperacillin/  tazobactam | 267 (8,7) | 204 (12,3) | 142 (6,2) | 214 (6,3) | 189 (5,5) | 204 (6,3) | 153 (4,7) | 128 (3,6) | 103 (2,9) | -0.9 (-1.3 to -0.5) ** | -5,8 |
|  | Piperacillin | 1744 (56,6) | 944 (57) | 1152 (50,6) | 1703 (50,4) | 1677 (49,1) | 1598 (49) | 1566 (47,8) | 1721 (47,8) | 1416 (40) | -1.7 (-2.2 to -1.1) *** | -16,6 |
|  | Cefotaxime | 504 (16,4) | 350 (21,1) | 357 (15,7) | 453 (13,4) | 465 (13,6) | 417 (12,8) | 425 (13) | 411 (11,4) | 354 (10) | -1.0 (-1.5 to -0.5) ** | -6,4 |
|  | Cefuroxime | 756 (24,5) | 499 (30,1) | 497 (21,8) | 618 (18,3) | 615 (18) | 567 (17,4) | 557 (17) | 543 (15,1) | 442 (12,5) | -1.7 (-2.4 to -1.1) ** | -12 |
|  | Ciprofloxacin | 862 (28) | 426 (25,7) | 622 (27,3) | 795 (23,5) | 797 (23,3) | 753 (23,1) | 647 (19,7) | 556 (15,5) | 441 (12,5) | -1.8 (-2.3 to -1.3) *** | -15,5 |
|  | Moxifloxacin | 1061 (34,4) | 564 (34,1) | 710 (31,2) | 953 (28,2) | 978 (28,6) | 846 (26) | 777 (23,7) | 817 (22,7) | 752 (21,3) | -1.7 (-1.9 to -1.5) *** | -13,1 |
|  | Levofloxacin | 821 (26,7) | 389 (23,5) | 578 (25,4) | 726 (21,5) | 721 (21,1) | 658 (20,2) | 570 (17,4) | 529 (14,7) | 424 (12) | -1.7 (-2.0 to -1.4) *** | -14,7 |
|  | Trimethoprim/  sulfamethoxazole | 1284 (41,7) | 698 (42,1) | 769 (33,8) | 1000 (29,6) | 1028 (30,1) | 930 (28,6) | 836 (25,5) | 942 (26,2) | 979 (27,7) | -2.0 (-2.8 to -1.2) ** | -14 |
|  | Imipenem | 2 (0,1) | 1 (0,1) | 0 (0) | 1 (0) | 0 (0) | 2 (0,1) | 2 (0,1) | 0 (0) | 1 (0) | 0.00 (-0.01 to 0.00) | -0,1 |
|  | Meropenem | 2 (0,1) | 0 (0) | 0 (0) | 0 (0) | 0 (0) | 0 (0) | 1 (0) | 0 (0) | 1 (0) | 0.00 (-0.01 to 0.00) | -0,1 |
| ***Enterococcus faecalis*** | **Total number of Isolates** | **1291** | **702** | **901** | **1161** | **1115** | **858** | **848** | **928** | **815** |  |  |
|  | Ampicillin* (Amp/Sul) | 8 (0,6) | 5 (0,7) | 2 (0,2) | 6 (0,5) | 12 (1,1) | 2 (0,2) | 8 (0,9) | 6 (0,6) | 10 (1,2) | +0.06 (-0.03 to 0.14) | +0,6 |
|  | Imipenem | 5 (0,4) | 2 (0,3) | 4 (0,4) | 14 (1,2) | 25 (2,2) | 7 (0,8) | 5 (0,5) | 8 (0,9) | 9 (1,1) | +0.5 (-0.4 to 1.4) | +0,7 |
|  | Vancomycin | 12 (0,9) | 2 (0,3) | 6 (0,7) | 9 (0,8) | 6 (0,5) | 7 (0,8) | 0 (0) | 4 (0,4) | 2 (0,2) | -0.1 (-0.1 to 0.0) | -0,7 |
| ***Enterococcus faecium*** | **Total number of Isolates** | **447** | **359** | **246** | **557** | **563** | **470** | **492** | **494** | **468** |  |  |
|  | Ampicillin/  sulbactam | 410 (92,6) | 310 (92,8) | 237 (96,3) | 507 (94,9) | 496 (93,6) | 429 (93,5) | 397 (90,6) | 442 (89,8) | 429 (91,9) | -0.4 (-0.9 to 0.0) | -0,7 |
|  | Imipenem | 414 (93,5) | 320 (96,1) | 240 (97,6) | 516 (96,3) | 505 (95,3) | 436 (95) | 452 (93,6) | 448 (90,9) | 432 (92,3) | -0.5 (-0.9 to -0.1) | -1,2 |
|  | Vancomycin | 147 (32,9) | 162 (45,1) | 93 (36,2) | 224 (40,3) | 228 (40,5) | 196 (41,7) | 221 (44,9) | 146 (29,6) | 130 (27,8) | -0.8 (-2.4 to 0.8) | -5,1 |
| ***Klebsiella pneumoniae*** | **Total number of Isolates** | **902** | **626** | **537** | **1006** | **1024** | **782** | **1005** | **1010** | **996** |  |  |
|  | Ampicillin/  sulbactam | 388 (43) | 248 (39,6) | 165 (30,7) | 335 (33,3) | 333 (32,6) | 274 (35) | 323 (35,2) | 254 (25,1) | 225 (22,6) | -1.9 (-2.9 to -0.9) ** | -20,4 |
|  | Ampicillin | 883 (97,9) | 611 (97,6) | 514 (95,7) | 965 (95,9) | 989 (96,7) | 761 (97,3) | 986 (98,1) | 968 (95,8) | 916 (92,1) | -0.4 (-0.8 to 0.0) | -5,8 |
|  | Cefotaxime | 219 (24,3) | 128 (20,4) | 87 (16,2) | 153 (15,2) | 157 (15,3) | 129 (16,5) | 165 (16,4) | 135 (13,4) | 146 (14,7) | -1.0 (-1.5 to -0.4) * | -9,6 |
|  | Cefuroxime | 366 (40,5) | 186 (29,7) | 121 (22,5) | 219 (21,8) | 235 (23) | 201 (25,7) | 228 (22,7) | 194 (19,2) | 200 (20,1) | -1.8 (-3.0 to -0.6) * | -20,4 |
|  | Ciprofloxacin | 254 (28,2) | 130 (20,8) | 100 (18,6) | 190 (18,9) | 201 (19,6) | 166 (21,2) | 186 (18,5) | 110 (10,9) | 138 (13,9) | -1.4 (-2.2 to -0.6) ** | -14,3 |
|  | Levofloxacin | 197 (21,8) | 72 (11,5) | 67 (12,5) | 114 (11,3) | 125 (12,2) | 94 (12) | 103 (10,2) | 81 (8) | 111 (11,2) | -0.9 (-1.7 to -0.2) * | -10,6 |
|  | Moxifloxacin | 352 (39) | 195 (31,2) | 134 (25) | 254 (25,3) | 279 (27,3) | 214 (27,4) | 256 (25,5) | 211 (20,9) | 243 (24,4) | -1.4 (-2.3 to -0.5) * | -14,6 |
|  | Trimethoprim/  sulfamethoxazole | 294 (32,6) | 143 (22,8) | 130 (24,2) | 201 (20) | 231 (22,6) | 181 (23,2) | 211 (21) | 164 (16,2) | 199 (20) | -1.2 (-2.0 to -0.4) * | -12,6 |
|  | Imipenem | 34 (3,8) | 11 (1,8) | 0 (0) | 12 (1,2) | 8 (0,8) | 2 (0,3) | 3 (0,3) | 4 (0,4) | 0 (0) | -0.3 (-0.5 to -0.1) * | -3,8 |
|  | Meropenem | 33 (3,7) | 10 (1,6) | 0 (0) | 7 (0,7) | 4 (0,4) | 1 (0,1) | 3 (0,3) | 0 (0) | 0 (0) | -0.3 (-0.5 to -0.1) * | -3,7 |
|  | Piperacillin/  tazobactam | 238 (26,4) | 163 (26) | 87 (16,2) | 176 (17,5) | 169 (16,5) | 165 (21,1) | 129 (12,8) | 128 (12,7) | 70 (7) | -2.0 (-2.9 to -1.2) ** | -19,4 |
|  | Piperacillin | 521 (57,8) | 347 (55,4) | 252 (46,9) | 523 (52) | 493 (48,2) | 420 (53,7) | 431 (42,9) | 353 (35) | 299 (30,1) | -3.0 (-4.2 to -1.7) ** | -27,7 |
| ***Pseudomonas aeruginosa*** | **Total number of Isolates** | **826** | **646** | **365** | **973** | **899** | **864** | **788** | **931** | **940** |  |  |
|  | Ceftazidime | 207 (25,1) | 142 (22) | 40 (11) | 119 (12,2) | 137 (15,2) | 139 (16,1) | 112 (14,2) | 89 (9,6) | 95 (10,1) | -1.4 (-2.4 to -0.5) * | -15 |
|  | Ciprofloxacin | 202 (24,5) | 183 (28,3) | 92 (25,2) | 240 (24,7) | 211 (23,5) | 227 (26,3) | 139 (17,6) | 124 (13,3) | 117 (12,4) | -1.8 (-2.6 to -0.9) ** | -12,1 |
|  | Levofloxacin | 284 (34,4) | 207 (32) | 87 (23,8) | 244 (25,1) | 214 (23,8) | 230 (26,6) | 168 (21,3) | 181 (19,5) | 168 (17,9) | -1.8 (-2.4 to -1.1) ** | -16,5 |
|  | Meropenem | 65 (7,9) | 54 (8,4) | 16 (5) | 50 (5,1) | 67 (7,4) | 39 (4,5) | 40 (5,1) | 41 (4,4) | 30 (3,2) | -0.5 (-0.8 to -0.2) * | -4,7 |
|  | Imipenem | 140 (16,9) | 136 (21,1) | 76 (20,8) | 257 (26,4) | 239 (26,5) | 208 (24,1) | 146 (18,5) | 135 (14,5) | 129 (13,7) | -0.7 (-1.9 to 0.5) | -3,2 |
|  | Piperacillin/  tazobactam | 212 (25,7) | 137 (21,2) | 53 (14,5) | 124 (12,8) | 115 (12,8) | 137 (15,9) | 118 (15) | 96 (10,3) | 84 (8,9) | -1.6 (-2.4 to -0.8) ** | -16,8 |
|  | Piperacillin | 261 (31,6) | 201 (31,1) | 80 (21,9) | 195 (20,1) | 189 (21,1) | 220 (25,5) | 157 (19,9) | 149 (16) | 118 (12,6) | -2.0 (-2.9 to -1.1) ** | -19 |
| ***Staph. aureus*** | **Total number of Isolates** | **1719** | **1742** | **1103** | **2342** | **2468** | **2270** | **2234** | **2329** | **2236** |  |  |
|  | Penicillin G | 1193 (69,3) | 1199 (68,9) | 847 (76,9) | 1360 (58,1) | 1471 (59,7) | 1358 (59,9) | 1303 (58,4) | 1279 (55,1) | 1213 (54,3) | -2.3 (-3.5 to -1.1) ** | -15 |
|  | Oxacillin | 291 (16,9) | 320 (18,4) | 496 (45) | 164 (7) | 193 (7,8) | 170 (7,5) | 134 (6) | 142 (6,1) | 105 (4,7) | -2.7 (-5.6 to 0.1) | -12,2 |
|  | Ciprofloxacin | 514 (29,9) | 516 (29,6) | 566 (51,3) | 458 (19,6) | 491 (19,9) | 442 (19,5) | 381 (17,1) | 366 (15,8) | 316 (14,1) | -2.9 (-5.2 to -0.6) * | -15,8 |
|  | Clindamycin | 193 (11,2) | 190 (10,9) | 216 (19,6) | 112 (4,8) | 141 (5,7) | 120 (5,3) | 99 (4,4) | 88 (3,8) | 83 (3,7) | -1.4 (-2.4 to -0.3) * | -7,5 |
|  | Levofloxacin | 490 (28,6) | 479 (27,5) | 551 (50) | 428 (18,3) | 465 (18,9) | 414 (18,2) | 352 (15,8) | 345 (14,9) | 291 (13) | -2.8 (-5.1 to -0.5) * | -15,6 |
|  | Moxifloxacin | 497 (28,9) | 489 (28,1) | 554 (50,3) | 428 (18,3) | 465 (18,9) | 416 (18,3) | 357 (16) | 343 (14,8) | 310 (13,9) | -2.8 (-5.1 to -0.5) * | -15 |
|  | Roxithromycin | 347 (20,2) | 334 (19,2) | 284 (25,7) | 309 (13,2) | 388 (15,7) | 399 (17,6) | 355 (15,9) | 403 (17,3) | 361 (16,2) | -0.6 (-1.5 to 0.2) | -4 |
|  | Trimethoprim/  sulfamethoxazole | 316 (18,5) | 350 (20,1) | 69 (6,3) | 154 (6,6) | 191 (7,8) | 150 (6,7) | 49 (2,2) | 10 (0,4) | 150 (6,7) | -1.9 (-3.0 to -0.8) * | -11,8 |
|  | Vancomycin | 2 (0,1) | 1 (0,1) | 2 (0,2) | 4 (0,2) | 1 (0) | 0 (0) | 1 (0) | 1 (0) | 1 (0) | -0.01 (-0.03 to 0.00) | -0,1 |
| ***Staph. epidermidis*** | **Total number of Isolates** | **1129** | **926** | **634** | **1189** | **1151** | **1246** | **1017** | **1134** | **1173** |  |  |
|  | Oxacillin | 908 (74,8) | 739 (79,9) | 437 (69) | 860 (72,7) | 863 (75,2) | 911 (73,2) | 719 (70,9) | 745 (65,8) | 759 (64,8) | -1.3 (-2.2 to -0.4) * | -10 |
|  | Ciprofloxacin | 810 (66,9) | 633 (68,5) | 410 (64,8) | 778 (65,6) | 770 (67) | 778 (62,4) | 599 (59,4) | 605 (54) | 629 (53,9) | -1.8 (-2.5 to -1.2) *** | -13 |
|  | Levofloxacin | 803 (66,5) | 630 (68,3) | 410 (65,3) | 766 (64,8) | 755 (65,9) | 760 (61,4) | 591 (58,5) | 610 (54,2) | 625 (53,5) | -1.9 (-2.4 to -1.3) *** | -13 |
|  | Moxifloxacin | 796 (65,7) | 624 (67,6) | 403 (64,2) | 753 (63,9) | 746 (65,2) | 743 (60,1) | 575 (56,7) | 591 (52,3) | 626 (53,6) | -1.9 (-2.5 to -1.3) *** | -12,1 |
|  | Clindamycin | 390 (34,5) | 421 (45,5) | 263 (41,5) | 525 (44,2) | 545 (47,4) | 539 (43,3) | 423 (41,6) | 468 (41,3) | 516 (44,1) | +0.4 (-0.5 to 1.3) | 9,6 |
|  | Trimethoprim/  sulfamethoxazole | 511 (42,4) | 392 (42,5) | 193 (30,7) | 347 (29,4) | 345 (30,2) | 339 (27,5) | 213 (21,1) | 165 (14,8) | 295 (25,5) | -2.9 (-4.0 to -1.7) ** | -16,9 |
|  | Roxithromycin | 778 (64,1) | 645 (69,8) | 402 (63,5) | 782 (65,9) | 769 (66,9) | 823 (66,1) | 634 (62,5) | 726 (64,1) | 752 (64,3) | -0.3 (-0.9 to 0.2) | +0,2 |
|  | Vancomycin | 3 (0,2) | 0 (0) | 0 (0) | 0 (0) | 2 (0,2) | 1 (0,1) | 0 (0) | 0 (0) | 1 (0,1) | -0.01 (-0.03 to 0.01) | -0,1 |

Legend: * = p < 0.05; ** = p < 0.01; *** = p < 0.001
