# Supplementary material for: Changes in antibiotic consumption, AMR and Clostridioides difficile infections in a large tertiary-care center following the implementation of institution-specific guidelines for antimicrobial therapy: A nine-year interrupted time series study
Source: PLoS One. 2021 Oct 14;16(10):e0258690. doi: 10.1371/journal.pone.0258690 (PMC8516227; doi:10.1371/journal.pone.0258690)
Supplement: S3 Table — (DOCX) [file pone.0258690.s004.docx]

**S3 Table.** Results of the interrupted time series analysis of changes in resistance rates in the LUH between 2012 and 2020 associated with the introduction of the ISGs.

| **Pathogen** | **Antibiotic agent** | **Baseline resistance rate in percent of total isolates (β0)** | **Baseline trend of resistance rate in percent of total isolates per month (β0)** | **Level change 1 month after the intervention in percent of total isolates(β2)** | **Trend change resistance rate after the intervention in percent per month (β3)** |
| --- | --- | --- | --- | --- | --- |
| ***E. coli*** | Cefotaxime | 18.4 (12.5 to 24.3) *** | -0.35 (-3.07 to 2.38) | -2.7 (-7.7 to 2.3) | -0.32 (-3.2 to 2.55) |
|  | Ciprofloxacin | 27.7 (22.5 to 32.9) *** | -0.35 (-2.78 to 2.08) | 1.2 (-3.3 to 5.7) | -2 (-4.57 to 0.56) |
|  | Cefuroxime | 28.2 (20.2 to 36.3) *** | -1.36 (-5.08 to 2.35) | -3.9 (-10.8 to 2.9) | 0.27 (-3.65 to 4.2) |
|  | Trimethoprim/sulfamethoxazole | 47.2 (41 to 53.3) *** | -3.99 (-6.84 to -1.14) ** | -4.8 (-10.1 to 0.4) | 3.29 (0.28 to 6.3) * |
|  | Ampicillin/sulbactam | 59.576 (49.144 to 70.008) *** | -4.897 (-9.726 to -0.068) * | 4.138 (-4.766 to 13.042) | 2.323 (-2.774 to 7.421) |
|  | Imipenem | 0.1 (0 to 0.2) * | -0.03 (-0.07 to 0.01) | 0 (-0.1 to 0.1) | 0.03 (-0.01 to 0.08) |
|  | Piperacillin/tazobactam | 11.5 (6 to 17.1) *** | -1.22 (-3.79 to 1.36) | -0.5 (-5.3 to 4.2) | 0.51 (-2.2 to 3.23) |
| ***Klebsiella pneumoniae*** | Ampicillin/sulbactam | 50.1 (38.9 to 61.2) *** | -6.14 (-11.31 to -0.98) * | 6.6 (-3 to 16.1) | 3.99 (-1.47 to 9.44) |
|  | Ciprofloxacin | 32.1 (23.5 to 40.6) *** | -0.048 (-0.087 to -0.008) * | 4.8 (-2.4 to 12.1) | 3.2 (-0.9 to 7.4) |
|  | Cefotaxime | 28.4 (25.2 to 31.6) *** | -4.04 (-5.53 to -2.55) *** | -0.2 (-2.9 to 2.6) | 3.79 (2.22 to 5.36) *** |
|  | Cefuroxime | 48.8 (42.7 to 55) *** | -8.97 (-11.83 to -6.1) *** | 2.4 (-2.9 to 7.7) | 8.32 (5.3 to 11.34) *** |
|  | Trimethoprim/sulfamethoxazole | 34.9 (26 to 43.9) *** | -4.19 (-8.34 to -0.04) * | 0.3 (-7.4 to 7.9) | 3.59 (-0.79 to 7.97) |
|  | Piperacillin/tazobactam | 33.1 (22.8 to 43.4) *** | -5.09 (-9.86 to -0.33) * | 4 (-4.8 to 12.8) | 3.03 (-2 to 8.07) |
|  | Imipenem | 5.6 (5 to 6.2) *** | -1.88 (-2.17 to -1.6) *** | 1.2 (0.7 to 1.8) *** | 1.68 (1.38 to 1.99) *** |
| ***Staph. aureus*** | Oxacillin | 15.4 (12.4 to 18.5) *** | 1.5 (-0.5 to 3.4) | -11.5 (-14.8 to -8.2) *** | -2 (-3.9 to 0) * |
|  | Penicillin G | 69.7 (62.5 to 76.8) *** | -0.4 (-4.9 to 4.1) | -7.5 (-15.2 to 0.2) | -0.6 (-5.2 to 4) |
|  | Vancomycin | 0.2 (-0.1 to 0.4) | -0.1 (-0.2 to 0.1) | 0.1 (-0.1 to 0.4) | 0 (-0.1 to 0.2) |
|  | Clindamycin | 11.5 (9.1 to 13.9) *** | -0.3 (-1.8 to 1.2) | -4.8 (-7.4 to -2.2) *** | 0 (-1.6 to 1.5) |
|  | Ciprofloxacin | 30.2 (26.3 to 34) *** | -0.3 (-2.7 to 2.2) | -7.5 (-11.7 to -3.3) *** | -0.9 (-3.4 to 1.6) |
|  | Trimethoprim/sulfamethoxazole | 16.8 (3.8 to 29.8) * | 1.7 (-6.6 to 9.9) | -14.2 (-28.3 to -0.1) * | -2.4 (-10.7 to 5.9) |
|  | Roxithromycin | 21.1 (15.1 to 27.2) *** | -1 (-4.8 to 2.9) | -4 (-10.6 to 2.5) | 1.5 (-2.4 to 5.4) |
| ***Staph. epidermidis*** | Oxacillin | 80.3 (69.8 to 90.9) *** | -2.88 (-7.75 to 2) | 5.8 (-3.2 to 14.8) | 0.87 (-4.28 to 6.01) |
|  | Vancomycin | 0.3 (0.1 to 0.6) ** | -0.12 (-0.24 to -0.01) * | 0.1 (-0.1 to 0.3) | 0.12 (0 to 0.24) |
|  | Clindamycin | 33.5 (23.6 to 43.4) *** | 3.5 (-1.08 to 8.09) | 1.6 (-6.8 to 10.1) | -4.09 (-8.93 to 0.76) |
|  | Ciprofloxacin | 68.8 (62.9 to 74.8) *** | -1.06 (-3.8 to 1.68) | 4.8 (-0.3 to 9.8) | -1.82 (-4.71 to 1.07) |
|  | Trimethoprim/sulfamethoxazole | 50.3 (35.5 to 65) *** | -5.85 (-12.67 to 0.96) | -0.7 (-13.3 to 11.8) | 3.79 (-3.4 to 10.99) |
|  | Roxithromycin | 66.4 (58.9 to 73.9) *** | -0.29 (-3.77 to 3.19) | 1.4 (-5 to 7.8) | -0.28 (-3.95 to 3.4) |
| ***Pseudomonas aeruginosa*** | Ciprofloxacin | 25.2 (16.3 to 34.2) *** | 0.38 (-3.75 to 4.51) | 3.3 (-4.3 to 10.9) | -3.24 (-7.6 to 1.12) |
|  | Piperacillin/tazobactam | 31.6 (24.9 to 38.3) *** | -5.57 (-8.67 to -2.47) *** | 0.5 (-5.3 to 6.2) | 4.79 (1.52 to 8.06) ** |
|  | Ceftazidim | 33.4 (25.5 to 41.3) *** | -7.05 (-10.71 to -3.39) *** | 3.6 (-3.2 to 10.3) | 6.2 (2.34 to 10.07) ** |
|  | Meropenem | 10 (6.3 to 13.6) *** | -1.44 (-3.15 to 0.26) | 1.2 (-2 to 4.3) | 0.92 (-0.88 to 2.72) |
|  | Imipenem | 15.7 (10.5 to 21) *** | 1.94 (-0.48 to 4.35) | 9.6 (5.1 to 14) *** | -4.94 (-7.49 to -2.39) *** |

Legend: * = p < 0.05; ** = p < 0.01; *** = p < 0.001
