# Supplementary material for: Changes in antibiotic consumption, AMR and Clostridioides difficile infections in a large tertiary-care center following the implementation of institution-specific guidelines for antimicrobial therapy: A nine-year interrupted time series study
Source: PLoS One. 2021 Oct 14;16(10):e0258690. doi: 10.1371/journal.pone.0258690 (PMC8516227; doi:10.1371/journal.pone.0258690)
Supplement: S4 Table — (DOCX) [file pone.0258690.s005.docx]

**S4 Table.** Annual German reference antibiotic resistance rates and trends for selected combinations of pathogens and antibiotic agents between 2012 and 2019, collected from the European Antimicrobial Resistance Network (EARS-Net). The data was accessed through the ECDC Surveillance Atlas for AMR (www.ecdc.europa.eu/en/antimicrobial-resistance/surveillance-and-disease-data/data-ecdc).

| **Pathogen** | **Antibiotic agent** | **2012 resistant isolates in percent** | **2013 resistant isolates in percent** | **2014 resistant isolates in percent** | **2015 resistant isolates in percent** | **2016 resistant isolates in percent** | **2017 resistant isolates in percent** | **2018 resistant isolates in percent** | **2019 resistant isolates in percent** | **Trend (simple linear regression)** |
| --- | --- | --- | --- | --- | --- | --- | --- | --- | --- | --- |
| ***Escherichia coli*** | 3G Cephalosporins | 8,84 | 10,74 | 10,53 | 10,30 | 11,10 | 12,28 | 12,20 | 11,51 | 0,381 (0,179 to 0,582) *** |
|  | Aminopenicillins | 49,59 | 52,92 | 51,72 | 49,37 | 48,97 | 48,94 | 49,23 | 48,66 | -0,401 (-0,789 to -0,014) * |
|  | Carbapenems | 0,02 | 0,09 | 0,06 | 0,03 | 0,01 | 0,01 | 0,02 | 0,01 | -0,008 (-0,015 to 0) |
|  | Fluoroquinolones | 21,11 | 22,07 | 20,59 | 19,38 | 19,45 | 20,75 | 19,83 | 17,53 | -0,425 (-0,717 to -0,133) ** |
| ***Klebsiella pneumoniae*** | 3G Cephalosporins | 12,95 | 16,06 | 12,72 | 10,25 | 13,62 | 14,61 | 12,94 | 12,23 | -0,139 (-0,684 to 0,407) |
|  | Fluoroquinolones | 13,73 | 15,08 | 12,65 | 9,56 | 12,65 | 15,32 | 13,35 | 13,13 | -0,021 (-0,602 to 0,561) |
| ***Pseudomonas aeruginosa*** | Carbapenems | 10,73 | 15,40 | 16,98 | 14,73 | 14,49 | 12,58 | 12,07 | 12,86 | -0,181 (-0,827 to 0,465) |
|  | Fluoroquinolones | 19,59 | 16,37 | 13,00 | 14,33 | 12,37 | 13,88 | 12,35 | 13,43 | -0,744 (-1,282 to -0,206) ** |
|  | Piperacillin/tazobactam | 12,74 | 17,44 | 15,09 | 15,98 | 15,04 | 12,55 | 12,41 | 11,70 | -0,488 (-1,032 to 0,056) |
| ***Staphylococcus aureus*** | Methicillin | 15,37 | 12,82 | 12,91 | 11,25 | 10,18 | 9,08 | 7,67 | 6,71 | -1,178 (-1,314 to -1,042) *** |

Legend: * = p < 0.05; ** = p < 0.01; *** = p < 0.001
